# Catalyst-free synthesis of 4-acyl-*NH*-1,2,3-triazoles by water-mediated cycloaddition reactions of enaminones and tosyl azide

**DOI:** 10.3762/bjoc.14.210

**Published:** 2018-09-07

**Authors:** Lu Yang, Yuwei Wu, Yiming Yang, Chengping Wen, Jie-Ping Wan

**Affiliations:** 1 College of Chemistry and Chemical Engineering, Jiangxi Normal University, Nanchang 330022, P. R. China; 2 College of Basic Medical Sciences, Zhejiang Chinese Medical University, Hangzhou 310053, P. R. China

**Keywords:** additive-free, catalyst-free, cycloaddition, enaminones, on water, 1,2,3-triazole

## Abstract

The synthesis of 4-acyl-*NH*-1,2,3-triazoles has been accomplished with high efficiency through the cycloaddition reactions between *N,N*-dimethylenaminones and tosyl azide. This method is featured with extraordinary sustainability by employing water as the sole medium, free of any catalyst or additive, authentically mild conditions (40 °C stirring) as well as practical scalability.

## Introduction

Discovering sustainable chemical syntheses constitutes one central issue of modern organic chemistry. A large number of strategies and concepts promoting sustainable syntheses have been conceived over the past decades. Methods employing water as reaction medium are amongst the most promising ones by avoiding the application of volatile organic solvents during the reaction process [1–3]. Besides acting as a safer and environmentally benign alternative to organic solvents, on water reactions are known for their accelerated reaction rates and improved synthetic selectivity [4–6]. Being inspired by these commonly recognized green features, flourishing advances in the research of water-mediated or promoted organic syntheses, including those reactions involving valuable C–C [7–11], C–heteroatom [12–16], heteroatom–heteroatom [17–18] bond formation as well as divergent cascade reactions [19–23], are presently taking place to guide the progress of sustainable organic synthesis.

1,2,3-Triazole is a heterocyclic moiety showing exceptionally broad and important applications as privileged structure in the discovery of biologically functional scaffolds, organic materials preparation, as directing group in transition-metal-catalyzed transformations and as key building block in the synthesis of numerous organic compounds [24–28]. The amazingly rapid and broad permeation of 1,2,3-triazoles to multidisciplinary areas can majorly be attributed to the occurrence of robust synthetic methods toward this heterocycle. The copper-catalyzed click [3 + 2] cycloaddition of azides and alkynes [29–32], for example, has served enormously to the advances in both the preparation and application of 1,2,3-triazoles. In addition, the discovery of other metal-catalyzed alkyne–azide cycloadditions (MAAC) providing 1,2,3-triazoles with diverse substitution patterns triggers the continuous development of these metal-catalyzed cycloaddition strategies [33–35]. Alongside the vast progress happened in MAAC-based 1,2,3-triazole synthesis, the past decade has witnessed the emergence of another powerful cycloaddition tool for the 1,2,3-triazole synthesis: the metal-free cycloaddition of azides with activated dipolarophiles. As synthetic tools being able to provide 1,2,3-triazoles using an organocatalyst or other non-metal catalysts, this method shows distinctive advantages in enabling the production of 1,2,3-triazoles free of any heavy metal contamination [36–38].

Generally, the cycloaddition of azides with activated dipolarophiles such as strained cyclic alkynes, enamines, enolates, electron-deficient olefins, ylides, iminium cations and alkyne anions, etc., have been identified as reliable approaches to access 1,2,3-triazole scaffolds with multiple substitution patterns [39–44]. In addition, the azide-free annulation has evolved also as another sustainable strategy for the synthesis of many 1,2,3-triazoles in the past decade [45–50]. More notably, besides occurring as active intermediate in the enamine-mediated cycloaddition for 1,2,3-triazole construction, enamines with good stability and easy availability such as enaminones have exhibited also conspicuously versatile application in the metal-free synthesis of divergent 1,2,3-triazoles by directly acting as starting materials [51–54]. In 2016, Dehaen and co-workers [55] reported the synthesis of N-substituted 1,2,3-triazoles via the reactions of organoazides and the in situ prepared *N,N*-dimethylenaminones by 150 °C microwave irradiation and subsequent heating in toluene at 100 °C, providing an effective protocol of enaminone-based 1,2,3-triazole synthesis. Interestingly, our continuous adventure in enaminone-based organic transformations has led us to the discovery that the cycloaddition of *N,N*-dimethylenaminones and tosyl azide efficiently affords *NH*-1,2,3-triazoles with water as the only medium, and not any catalyst or additive is required. Considering the featured functions of *NH*-1,2,3-triazoles [56–61] as well as the urgent desire in finding more sustainable methods enabling 1,2,3-triazole synthesis, we report herein our results in the water-mediated, catalyst-free synthesis of *NH*-1,2,3-triazoles through the cycloaddition of enaminone and sulfonyl azide with mild heating (40 °C) and simple operation.

## Results and Discussion

To start the work, the reaction of enaminone **1a** and tosyl azide (**2**) was tentatively run in water by heating at 60 °C in the presence of *t*-BuONa, which provided *NH*-1,2,3-triazole product **3a** with 52% yield together with *N,N*-dimethyl tosyl amide as byproduct (entry 1, Table 1). Varying the additive to AcOH didn’t lead to an improved result (entry 2, Table 1). To our delight, the parallel entry without using any catalyst or additive afforded **3a** with identically good yield (entry 3, Table 1). With this encouraging result, we then carried out a systematic screen of the reaction parameters using water as the fixed reaction medium. First, a slight increase in the loading of tosyl azide was able to evidently enhance the yield of **3a** (entries 4 and 5, Table 1). Furthermore, the examination on the impact of the reaction temperature led to the observation of an excellent product yield by running the reaction at 40 °C (entries 6–8, Table 1). The variation on the volume of the water, on the other hand, gave no better reaction results (entries 9 and 10, Table 1). Finally, a control experiment employing EtOH as the reaction medium gave **3a** with evidently lower yield than the equivalent reaction using water (entry 11, Table 1).

**Table 1:** Screen and optimization of the reaction conditions.^a^

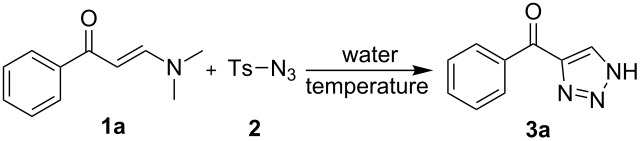			

entry	*T* (°C)	additive	yield (%)^b^

1	60	*t*-BuONa	52
2	60	AcOH	45
3	60	–	52
4^c^	60	–	75
5^d^	60	–	76
6^c^	80	–	80
7^c^	100	–	72
8^c^	40	–	89
9^c,e^	40	–	83
10^c,f^	40	–	83
11^c,g^	40	–	22

^a^General conditions: enaminone **1a** (0.2 mmol), tosyl azide (**2**, 0.2 mmol), additive (1 equiv) were stirred for 20 h in water (2.0 mL). ^b^Yield of isolated product based on **1a**. ^c^0.3 mmol **2**. ^d^0.24 mmol **2**. ^e^H_2_O (3 mL) was used. ^f^H_2_O (1 mL) was used. ^g^EtOH was used as alternative reaction medium.

**Figure 1:**
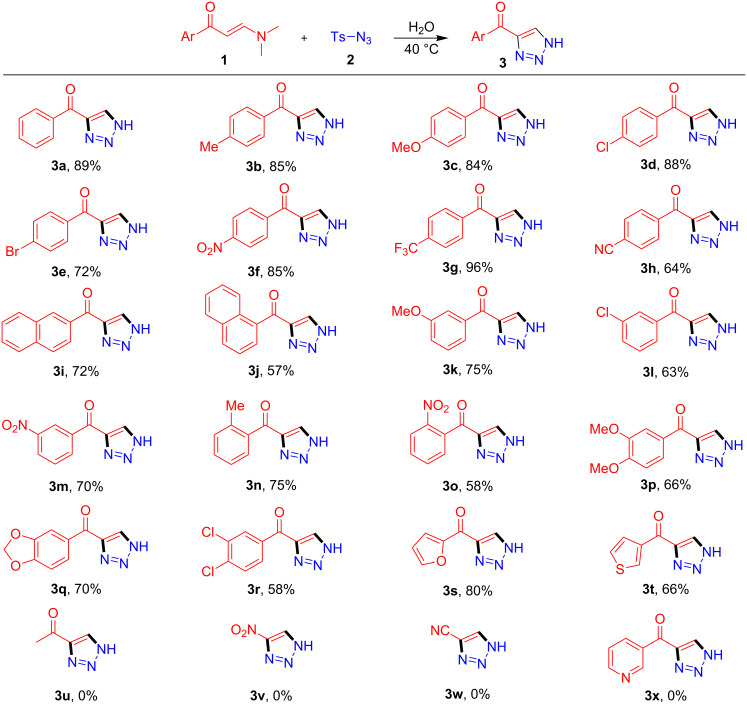
Scope of the water-mediated synthesis of 4-acyl-*NH*-1,2,3-triazoles. General conditions: enaminone **1** (0.2 mmol), tosyl azide **2** (0.3 mmol) and water (2 mL), stirred at 40 °C for 20 h (yields of isolated products are based on **1**).

To examine the scope of this water-mediated 1,2,3-triazole synthesis, a broad range of enaminones **1** was then employed to react with tosyl azide under the optimal conditions. According to the acquired results (Figure 1), satisfactory tolerance of this water-mediated, catalyst-free protocol was verified by the smooth synthesis of the 4-acyl-*NH*-1,2,3-triazoles **3a**–**t** containing versatile substructures (Figure 1). Besides the successful reactions employing enaminones independently containing electron-withdrawing and donating groups in the phenyl ring (H, alkyl, alkoxyl, halogen, CF_3_ and cyano, etc.), the substitution in *ortho-* (**3n**, **3o**, Figure 1) and *meta*-position of the phenyl ring (**3k**–**m**, Figure 1) were also readily compatible with the synthesis. More notably, those enaminones functionalized with disubstituted phenyls (**3p**–**r**, Table 2) as well as heteroaryl-based enaminones (**3s** and **3t**, Figure 1) also participated in the reaction to provide the divergently functionalized *NH*-1,2,3-triazoles. The products were generally furnished with good to excellent yield, and the variation of product yields was found to associate with both the electron property and the sites of the substituent in the aryl ring of **1**. However, when methyl-functionalized enaminone, *N,N*-dimethyl nitroenaminone, *N,N*-dimethyl cyanoenaminone, or pyridine-3-yl-functionalized enaminone was individually utilized, the expected reaction did not take place (**3u**–**x**, Figure 1). Moreover, it is notable that no *N*-sulfonyl-1,2,3-triazole was isolated from any of the above experiments, indicating the excellent chemoselectivity of the present synthetic method.

**Scheme 1:**
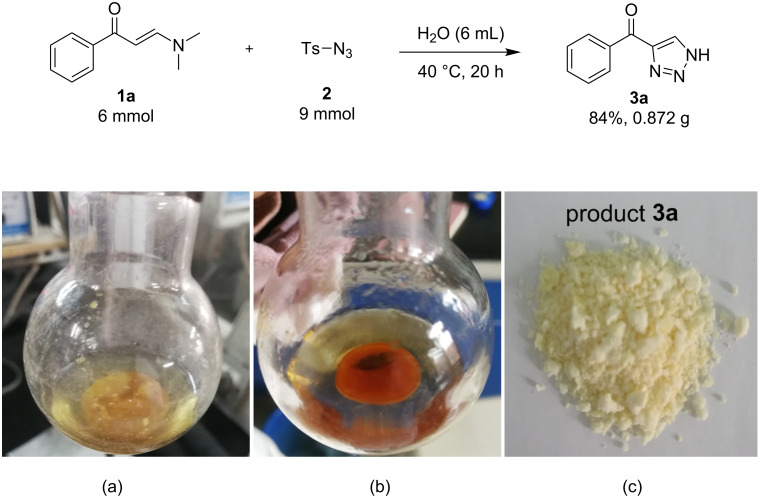
The gram scale synthesis of **3a**: (a) before reaction; (b) completed reaction; (c) the purified product **3a**.

In order to illustrate the potential application of this authentically green synthetic method, a gram scale synthesis of product **3a** was conducted starting from enaminone **1a** and tosyl azide (**2**). As expected, this entry turned out to be highly efficient affording product **3a** with excellent yield (Scheme 1). In addition, the appearance of the reaction mixture before and after the reaction indicated the reaction as a heterogeneous “on water” process (Scheme 1).

**Scheme 2:**
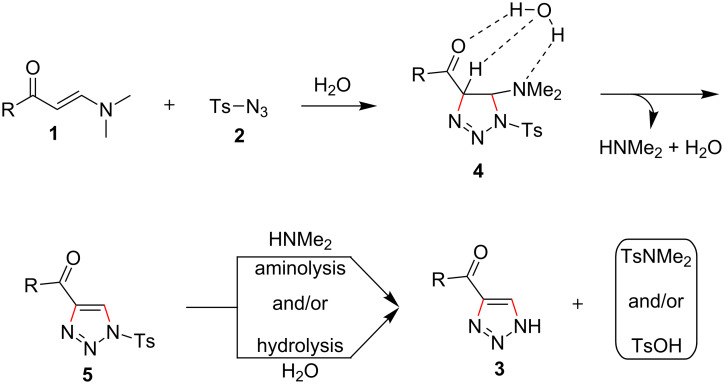
The proposed reaction mechanism.

Based on the known works employing organic solvents for similar synthesis and the present results [55], a possible mechanism for the reaction is proposed (Scheme 2). The reaction starts from the cycloaddition of enaminones **1** and tosyl azide (**2**) to provide 1,2,3-triazoline **4** which couples to water by strong hydrogen bond effect [51]. The presence of the hydrogen bonds may promote the elimination of the amino group and the acidic C–H bond at the α-position of the acyl group, which affords *N*-tosyl-1,2,3-triazole **5**. Under the present reaction conditions, the intermediate **5** can undergo aminolysis and/or hydrolysis to provide the target products **3**. The participation of water throughout the reaction also explains the high efficiency of the method using water as reaction medium.

## Conclusion

In summary, by means of the cycloaddition reactions between tertiary enaminones and tosyl azide employing water the sole reaction medium, a series of 4-acyl-*NH*-1,2,3-triazoles has been efficiently synthesized under catalyst-free and very mild heating conditions, thus providing the first water-mediated metal-free method toward the synthesis of 4-acyl-*NH*-1,2,3-triazoles. The present method benefits from unique sustainability not only due to the metal/additive-free cycloaddition reaction, but also by applying the completely green reaction medium water and mild reaction temperature.

## Supporting Information

File 1 General experimental information, experimental details of the synthesis of products **3**, full characterization data as well as ^1^H/^13^C NMR spectra of all products.

## References

[1] Lipshutz B H, Ghorai S, Cortes-Clerget M (2018). Chem – Eur J.

[2] Simon M-O, Li C-J (2012). Chem Soc Rev.

[3] Anastas P, Eghbali N (2010). Chem Soc Rev.

[4] Gawande M B, Bonifácio V D B, Luque R, Branco P S, Varma R S (2013). Chem Soc Rev.

[5] Butler R N, Goyne A G (2010). Chem Rev.

[6] Chanda A, Fokin V V (2009). Chem Rev.

[7] Guo W, Wu B, Zhou X, Chen P, Wang X, Zhou Y-G, Liu Y, Li C (2015). Angew Chem, Int Ed.

[8] Li Y, Huang Y, Gui Y, Sun J, Li J, Zha Z, Wang Z (2017). Org Lett.

[9] Zhang F-Z, Tian Y, Li G-X, Qu J (2015). J Org Chem.

[10] Álvarez M, Gava R, Rodríguez M R, Rull S G, Pérez P J (2017). ACS Catal.

[11] Zhang N, Yang D, Wei W, Yuan L, Nie F, Tian L, Wang H (2015). J Org Chem.

[12] Xie L-Y, Li Y-J, Qu J, Duan Y, Hu J, Liu K-J, Cao Z, He W-M (2017). Green Chem.

[13] Xiao F, Chen S, Tian J, Huang H, Liu Y, Deng G-J (2016). Green Chem.

[14] Liu K-J, Fu Y-L, Xie L-Y, Wu C, He W-B, Peng S, Wang Z, Bao W-H, Cao Z, Xu X (2018). ACS Sustainable Chem Eng.

[15] Wu C, Xin X, Fu Z-M, Xie L-Y, Liu K-J, Wang Z, Li W, Yuan Z-H, He W-M (2017). Green Chem.

[16] Li W, Yin G, Huang L, Xiao Y, Fu Z, Xin X, Liu F, Li Z, He W (2016). Green Chem.

[17] Tang L, Yang Y, Wen L, Yang X, Wang Z (2016). Green Chem.

[18] Lin Y-m, Lu G-p, Wang G-x, Yi W-b (2017). J Org Chem.

[19] Köhling S, Exner M P, Nojoumi S, Schiller J, Budisa N, Rademann J (2016). Angew Chem, Int Ed.

[20] Chen D, Feng Q, Yang Y, Cai X-M, Wang F, Huang S (2017). Chem Sci.

[21] Yang J, Mei F, Fu S, Gu Y (2018). Green Chem.

[22] Liu J, Lei M, Hu L (2012). Green Chem.

[23] Reddy G T, Kumar G, Reddy N C G (2018). Adv Synth Catal.

[24] Kolb H C, Sharpless K B (2003). Drug Discovery Today.

[25] Nandivada H, Jiang X, Lahann J (2007). Adv Mater.

[26] Thirumurugan P, Matosiuk D, Jozwiak K (2013). Chem Rev.

[27] Chen Z, Liu Z, Gao G, Li H, Ren H (2017). Adv Synth Catal.

[28] Xie L-Y, Qu J, Peng S, Liu K-J, Wang Z, Ding M-H, Wang Y, Cao Z, He W-M (2018). Green Chem.

[29] Huisgen R (1963). Angew Chem, Int Ed Engl.

[30] Kolb H C, Finn M G, Sharpless K B (2001). Angew Chem, Int Ed.

[31] Tornøe C W, Christensen C, Meldal M (2002). J Org Chem.

[32] Hein J E, Fokin V V (2010). Chem Soc Rev.

[33] Zhang L, Chen X, Xue P, Sun H H Y, Williams I D, Sharpless K B, Fokin V V, Jia G (2005). J Am Chem Soc.

[34] Destito P, Couceiro J R, Faustino H, López F, Mascareñas J L (2017). Angew Chem, Int Ed.

[35] Johansson J R, Lincoln P, Nordén B, Kann N (2011). J Org Chem.

[36] Ramasastry S S V (2014). Angew Chem, Int Ed.

[37] Lima C G S, Ali A, van Berkel S S, Westermann B, Paixão M W (2015). Chem Commun.

[38] Thomas J, Jana S, John J, Liekens S, Dehaen W (2016). Chem Commun.

[39] Ramachary D B, Shashank A B, Karthik S (2014). Angew Chem, Int Ed.

[40] Li W, Wang J (2014). Angew Chem, Int Ed.

[41] Agard N J, Preschner J A, Bertozzi C R (2004). J Am Chem Soc.

[42] Ramachary D B, Ramakumar K, Narayana V V (2008). Chem – Eur J.

[43] Belkheira M, Abed D E, Pons J-M, Bressy C (2011). Chem – Eur J.

[44] Kwok S W, Fotsing J R, Fraser R J, Rodionov V O, Fokin V V (2010). Org Lett.

[45] Wan J-P, Hu D, Liu Y, Sheng S (2015). ChemCatChem.

[46] Chen Z, Cao G, Song J, Ren H (2017). Chin J Chem.

[47] van Berkel S S, Brauch S, Gabriel L, Henze M, Stark S, Vasilev D, Wessjohann L A, Abbas M, Westermann B (2012). Angew Chem, Int Ed.

[48] Chen Z, Yan Q, Liu Z, Zhang Y (2014). Chem – Eur J.

[49] Cai Z-J, Lu X-M, Zi Y, Yang C, Shen L-J, Li J, Wang S-Y, Ji S-J (2014). Org Lett.

[50] Wan J-P, Cao S, Liu Y (2015). J Org Chem.

[51] Bakulev V A, Beryozkina T, Thomas J, Dehaen W (2018). Eur J Org Chem.

[52] Cheng G, Zeng X, Shen J, Wang X, Cui X (2013). Angew Chem, Int Ed.

[53] Wan J-P, Cao S, Liu Y (2016). Org Lett.

[54] Efimov I, Bakulev V, Beliaev N, Beryozkina T, Knippschild U, Leban J, Zhi-Jin F, Eltsov O, Slepukhin P, Ezhikova M (2014). Eur J Org Chem.

[55] Thomas J, Goyvaerts V, Liekens S, Dehaen W (2016). Chem – Eur J.

[56] Thomas J, Jana S, Liekens S, Dehaen W (2016). Chem Commun.

[57] Cohrt A E, Jensen J F, Nielsen T E (2010). Org Lett.

[58] Hu Q, Liu Y, Deng X, Li Y, Chen Y (2016). Adv Synth Catal.

[59] Qvortrup K, Nielsen T E (2011). Chem Commun.

[60] Deng X, Lei X, Nie G, Jia L, Li Y, Chen Y (2017). J Org Chem.

[61] Bakulev V A, Beryozkina T A (2016). Chem Heterocycl Compd.

