# Supplementary material for: Catalyst-free synthesis of 4-acyl-NH-1,2,3-triazoles by water-mediated cycloaddition reactions of enaminones and tosyl azide
Source: Beilstein J Org Chem. 2018 Sep 7;14:2348–53. doi: 10.3762/bjoc.14.210 (PMC6142726; doi:10.3762/bjoc.14.210)
Supplement: File 1 — General experimental information, experimental details of the synthesis of products 3, full characterization data as well as 1H/13C NMR spectra of all products. [file Beilstein_J_Org_Chem-14-2348-s001.pdf]

Supporting Information
for
Catalyst-free synthesis of 4-acyl-*NH*-1,2,3-triazoles by water-mediated cycloaddition reactions of enaminones and tosyl azide

Lu Yang[1], Yuwei Wu[1], Yiming Yang[1], Chengping Wen*[2], and Jie-Ping Wan*[1]

Address: [1]College of Chemistry and Chemical Engineering, Jiangxi Normal University, Nanchang 330022, P. R. China and [2]College of Basic Medical Sciences, Zhejiang Chinese Medical University, Hangzhou 310053, P. R. China

Email: Jie-Ping Wan - wanjieping@jxnu.edu.cn; Chengping Wen - cpwen.zcmu@yahoo.com

*Corresponding author

General experimental information, experimental details of the synthesis of products 3, full characterization data as well as ^{1}H/^{13}C NMR spectra of all products

General experimental information

All experiments were carried out at open atmosphere and the reactions are monitored by TLC (GF254 silica gel). Enaminones were synthesized following literature procedures,[1] other chemicals and solvents were obtained from commercial sources and used directly without further treatment. ^{1}H And ^{13}C NMR spectra were recorded with a spectrometer operating at 400 MHz (^{1}H) or 100 MHz (^{13}C), respectively. The chemical shifts of most of compounds were reported in ppm with TMS as internal standard. Melting points were determined with an X-4A instrument without correcting temperature and the HRMS were obtained using ESI in an apparatus equipped with a TOF analyzer.

General procedure for the synthesis of 1,2,3-triazoles 3. In a 25 mL round-bottomed flask equipped with a condenser was added enaminone **1** (0.2 mmol), tosyl azide (**2**, 0.3 mmol) and H_2O (2.0 mL). Then the mixture was stirred at 40 °C for 20 h. Upon completion (TLC), the reaction mixture was allowed to cool to room temperature and diluted with water (5 mL). The resulting suspension was extracted with ethyl acetate (3 × 8 mL). The combined organic solution was dried over anhydrous Na_2SO_4. After filtration, the solvent was removed at reduced pressure. Purification of the residue by flash column chromatography using mixed ethyl acetate (EA) and petroleum ether (PE) as eluent (EA/PE 1:3) afforded analytically pure product.

Procedure for the scale up synthesis of 1,2,3-triazole 3a. Enaminone **1a** (6 mmol, 1.086 g), tosyl azide **2** (9 mmol, 1.773 g; caution: tosyl azide is potentially explosive,

heavy shaking or high temperature should be avoided in large scale operation) and water (6 mL) were placed in a 25 mL round-bottomed flask. The mixture was heated at 40 °C under stirring for 20 h. After cooling to room temperature, 10 mL water were added, and the resulting suspension extracted with ethyl acetate (3 × 20 mL). The organic phases were combined and dried with anhydrous Na_2SO_4. After filtration, the solution was subjected to reduced pressure to remove the solvent. Purification of the residue by flash column chromatography using mixed ethyl acetate (EA) and petroleum ether (PE) as eluent (EA/PE 1:3) afforded **3a** with 84% yield (0.872 g).

Characterization data of all products

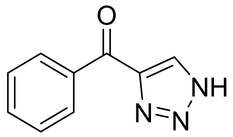

Phenyl(1*H*-1,2,3-triazol-4-yl)methanone (**3a**).[2] Yield: 31 mg (89 %); white solid, m.p. 169-170 °C; ^{1}H NMR (400 MHz, $CDCl_3$): δ 8.41 (s, 1H), 8.28 (d, *J* = 6.4 Hz, 2H), 7.65 (d, *J* = 6.8 Hz, 1H), 7.54 (t, *J* = 6.4 Hz, 2H); ^{13}C NMR (100 MHz, $CDCl_3$): δ 186.1, 136.5, 134.0, 133.7, 130.2, 128.6.

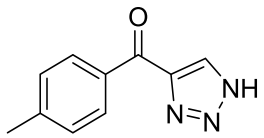

***p*-Tolyl(1*H*-1,2,3-triazol-4-yl)methanone** (**3b**). Yield: 32 mg (85 %); white solid, m.p. 98 °C ; ^{1}H NMR (400 MHz, DMSO-d_6): δ 8.65 (s, 1H), 8.15 (d, *J* = 8.0 Hz, 2H), 7.38 (d, *J* = 8.0 Hz, 2H), 2.40 (s, 3H); ^{13}C NMR (100 MHz, DMSO-d_6): δ 185.4, 144.2, 134.5, 132.4, 130.5, 129.6, 21.7; ESI-HRMS: Calcd for $C_{10}H_8N_3O$ $[M+H]^+$ 188.0818, found188.0819.

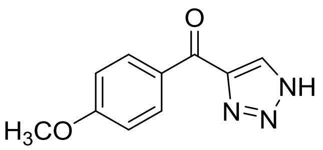

(4-Methoxyphenyl)(1*H*-1,2,3-triazol-4-yl)methanone (**3c**). Yield: 34 mg (84 %); white solid, m.p. 128 °C; ¹H NMR (400 MHz, $CDCl_3$): δ 8.34 (s, 1H), 8.26 (d, *J* = 8.8 Hz, 2H), 6.92 (d, *J* = 8.8 Hz, 2H), 3.81 (s, 3H); ¹³C NMR (100 MHz, $CDCl_3$): δ 184.5, 164.2, 133.7, 132.8, 129.3, 113.9, 55.6; ESI-HRMS: Calcd for $C_{10}H_9N_3O_2$ $[M+H]^+$ 204.0768, found 204.0766.

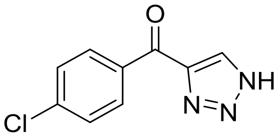

(**4-Chlorophenyl)(1*H*-1,2,3-triazol-4-yl)methanone** (**3d**). Yield: 36 mg (88 %); white solid, m.p. 196 °C; ¹H NMR (400 MHz, DMSO-*d*₆): δ 8.76 (s, 1H), 8.30 (d, *J* = 8.4 Hz, 1H), 7.69 (d, *J* = 8.4 Hz, 1H); ¹³C NMR (100 MHz, DMSO-*d*₆): δ 184.7, 138.7, 135.7, 132.3, 129.2; ESI-HRMS: Calcd for $C_9H_6ClN_3O$ $[M+H]^+$ 208.0272, found 208.0273.

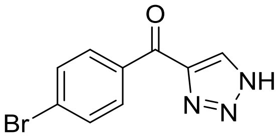

(4-Bromophenyl)(1*H*-1,2,3-triazol-4-yl)methanone (**3e**). Yield: 36 mg (72 %); yellow liquid; ¹H NMR (400 MHz, DMSO-*d*₆): δ 8.72 (s, 1H), 8.18 (d, *J* = 8.0 Hz, 2H), 7.79 (d, *J* = 8.4 Hz, 2H); ¹³C NMR (100 MHz, DMSO-*d*₆): δ 185.0, 146.1, 136.0, 132.4, 132.1, 127.9; ESI-HRMS: Calcd for $C_9H_6BrN_3O$ $[M+H]^+$251.9767, found251.9768.

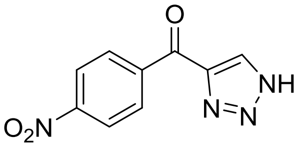

(4-Nitrophenyl)(1*H*-1,2,3-triazol-4-yl)methanone (**3f**). Yield: 37 mg (85 %); yellow solid, m.p. 169 °C; ¹H NMR (400 MHz, DMSO-*d*₆): δ 8.81 (s, 1H), 8.45-8.37 (m, 4H); ¹³C NMR (100 MHz, DMSO): δ 184.8, 150.3, 145.8, 142.0, 131.7, 124.0; ESI-HRMS: Calcd for $C_9H_6N_4O_3$ $[M+H]^+$ 219.0513, found 219.0515.

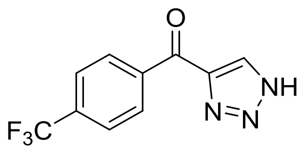

(1*H*-1,2,3-Triazol-4-yl)(4-(trifluoromethyl)phenyl)methanone (**3g**). Yield: 46 mg (96 %); white solid, m.p. 135 °C; ^{1}H NMR (400 MHz, DMSO-*d*$_6$) δ 16.0 (brs, 1H), 8.79 (s, 1H), 8.39 (d, *J* = 8.0 Hz, 2H), 7.95 (d, *J* = 2.8 Hz, 2H); ^{13}C NMR (100 MHz, DMSO-*d*$_6$): δ 185.2, 145.8, 140.5, 132.7 (q, $J_{C\text{-}F}$ = 32.4 Hz), 131.1, 125.9 (q, *J* = 3.8 Hz), 125.6, 122.9; ESI-HRMS: Calcd for $C_{10}H_6F_3N_3O$ [M+H]$^+$ 242.0536, found 242.0538.

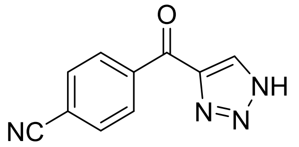

4-(1*H*-1,2,3-Triazol-4-carbonyl)benzonitrile (**3h**). Yield: 25 mg (64 %); white solid, m.p. 159 °C; ^{1}H NMR (400 MHz, DMSO-*d*$_6$): δ 8.81 (s, 1H), 8.35 (d, *J* = 8.4 Hz, 2H), 8.08 (d, *J* = 8.4 Hz, 2H); ^{13}C NMR (100 MHz, DMSO-*d*$_6$): δ 185.1, 145.8, 140.6, 133.0, 130.9, 118.7, 115.6; ESI-HRMS: Calcd for $C_{10}H_5N_4O$ [M+H]$^+$ 199.0614, found 199.0618.

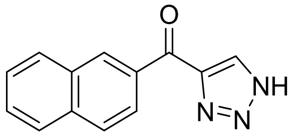

Naphthalen-2-yl(1*H*-1,2,3-triazol-4-yl)methanone (**3i**). Yield: 32 mg (72 %); white solid, m.p. 168 °C; ^{1}H NMR (400 MHz, DMSO-*d*$_6$): δ 8.96 (s, 1H), 8.76 (s, 1H), 8.16 (t, *J* = 9.6 Hz, 2H), 8.07 (d, *J* = 8.4 Hz, 1H), 8.01 (d, *J* = 8.0 Hz, 1H), 7.68 (t, *J* = 7.6 Hz, 1H), 7.62 (t, *J* = 7.6 Hz, 1H); ^{13}C NMR (100 MHz, DMSO-*d*$_6$): δ 185.9, 146.1, 135.5, 134.4, 132.6, 132.4, 130.3, 129.3, 128.7, 128.1, 127.4, 125.6; ESI-HRMS: Calcd for $C_{13}H_9N_3O$ [M+H]$^+$224.0818, found 224.0817.

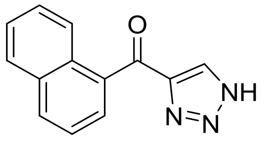

Naphthalen-1-yl(1*H*-1,2,3-triazol-4-yl)methanone (**3j**). Yield: 25 mg (57 %); yellow liquid; ^{1}H NMR (400 MHz, DMSO-*d*$_6$): δ 8.71 (s, 1H), 8.20 (d, *J* = 8.0 Hz,

2H), 8.11-8.04 (m, 1H), 7.96 (d, J = 7.2 Hz, 1H), 7.69-7.59 (m, 3H); ^{13}C NMR (100 MHz, DMSO-d_6): δ 189.0, 135.5, 133.8, 132.4, 130.4, 129.6, 129.0, 128.0, 126.9, 125.4, 125.2; ESI-HRMS: Calcd for $C_{13}H_9N_3O$ [M+H]$^+$ 224.0818, found 224.815.

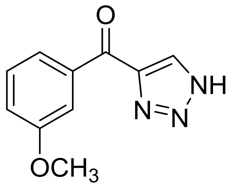

(3-Methoxyphenyl)(1*H*-1,2,3-triazol-4-yl)methanone (**3k**). Yield: 30 mg (75 %); white solid, m.p. 138 ^{o}C; ^{1}H NMR (400 MHz, DMSO-d_6): δ 8.72 (s, 1H), 7.87 (d, J = 7.2 Hz, 1H), 7.76 (s, 1H), 7.54 (d, J = 7.6 Hz, 1H), 7.30 (d, J = 8.0 Hz, 1H), 3.87 (s, 3H); ^{13}C NMR (100 MHz, DMSO-d_6): δ 185.8, 159.6, 146.1, 138.4, 130.2, 122.9, 119.7, 114.9, 55.8; ESI-HRMS: Calcd for $C_{10}H_9N_3O_2$ [M+H]$^+$ 204.0768, found 204.0767.

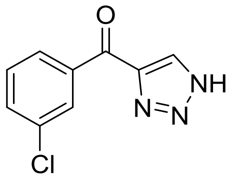

(3-Chlorophenyl)(1*H*-1,2,3-triazol-4-yl)methanone (**3l**). Yield: 30 mg (63 %); white solid, m.p. 98 ^{o}C; ^{1}H NMR (400 MHz, $CDCl_3$) δ 8.41 (s, 1H), 8.15 (s, 1H), 8.08 (d, J = 7.6 Hz, 1H), 7.51 (d, J = 8.0 Hz, 1H), 7.38 (d, J = 7.8 Hz, 1H); ^{13}C NMR (100 MHz, $CDCl_3$) δ 184.9, 145.6, 137.9, 134.9, 133.6, 130.1, 130.0, 128.4; ESI-HRMS: Calcd for $C_9H_6ClN_3O$ [M+H]$^+$ 208.0272, found 208.0277.

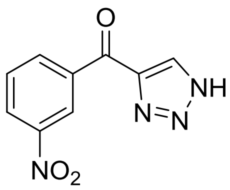

(3-Nitrophenyl)(1*H*-1,2,3-triazol-4-yl)methanone (**3m**). Yield: 30 mg (70 %); yellow liquid; ^{1}H NMR (400 MHz, DMSO-d_6): δ 9.07 (s, 1H), 8.82 (s, 1H), 8.63 (d, J = 7.6 Hz, 1H), 8.51 (d, J = 8.4 Hz, 1H), 7.88 (d, J = 8.0 Hz, 1H); ^{13}C NMR (100 MHz, DMSO-d_6): δ 183.7, 148.2, 146.0, 138.0, 136.4, 130.8, 127.9, 125.1; ESI-HRMS: Calcd for $C_9H_6N_4O_3$ [M+H]$^+$ 219.0513, found 219.0513.

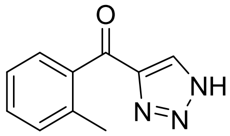

***o*-Tolyl(1*H*-1,2,3-triazol-4-yl)methanone** (**3n**). Yield: 28 mg (75 %); white solid, m.p. 139 °C; ^{1}H NMR (400 MHz, DMSO-*d*$_6$): δ 8.59 (s, 1H), 7.61 (d, *J* = 7.6 Hz, 1H), 7.48 (d, *J* = 7.6 Hz, 1H), 7.38-7.31 (m, 2H), 2.33 (s, 3H); ^{13}C NMR (100 MHz, DMSO-*d*$_6$): δ 183.0, 138.3, 137.0, 131.5, 131.4, 129.8, 129.3, 125.8, 20.2; ESI-HRMS: Calcd for $C_{10}H_9N_3O$ [M+H]$^+$ 188.0818, found 188.0819.

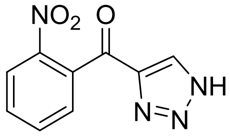

(2-Nitrophenyl)(1*H*-1,2,3-triazol-4-yl)methanone (**3o**). Yield: 25 mg (58 %); yellow liquid; ^{1}H NMR (400 MHz, CDCl$_3$): δ 8.31 (s, 1H), 8.15 (d, *J* = 8.0 Hz, 1H), 7.76 (d, *J* = 7.6 Hz, 1H), 7.66 (d, *J* = 7.8 Hz, 1H), 7.58 (d, *J* = 7.2 Hz, 1H); ^{13}C NMR (100 MHz, CDCl$_3$): δ 186.1, 147.1, 145.9, 134.7, 134.4, 131.5, 129.1, 124.3; ESI-HRMS: Calcd for $C_9H_6N_4O_3$ [M+H]$^+$ 219.0513, found 219.0516.

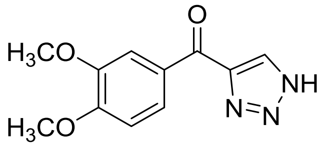

(3,4-Dimethoxyphenyl)(1*H*-1,2,3-triazol-4-yl)methanone (**3p**). Yield: 31 mg (66 %); white solid, m.p. 204 °C; ^{1}H NMR (400 MHz, DMSO-*d*$_6$): δ 8.63 (s, 1H), 8.07 (s, 1H), 7.78 (s, 1H), 7.16 (d, *J* = 8.0 Hz, 1H), 3.88 (s, 3H), 3.84 (s, 3H); ^{13}C NMR (100 MHz, DMSO-*d*$_6$): δ 184.3, 153.8, 149.0, 146.6, 129.6, 128.6, 125.7, 112.5, 111.4, 56.3, 56.0; ESI-HRMS: Calcd for $C_{11}H_{11}N_3O_3$ [M+H]$^+$ 234.0873, found 234.0870.

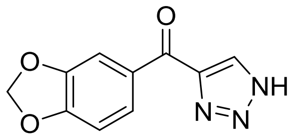

Benzo[*d*][1,3]dioxol-5-yl(1*H*-1,2,3-triazol-4-yl)methanone (**3q**). Yield: 30 mg (70 %); white solid, m.p. 182 °C; ^{1}H NMR (400 MHz, DMSO-*d*$_6$): δ 15.8 (brs, 1H), 8.75 (s, 1H), 8.03 (s, 1H), 7.73 (s, 1H), 7.12 (d, *J* = 9.6 Hz, 1H), 6.18 (s, 2H); ^{13}C

NMR (100 MHz, DMSO-d_6): δ 183.5, 152.2, 148.1, 146.6, 131.3, 127.4, 109.5, 108.6, 102.6; ESI-HRMS: Calcd for $C_{10}H_7N_3O_3$ $[M+H]^+$ 218.0560, found 218.0559.

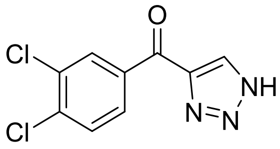

(3,4-Dichlorophenyl)(1*H*-1,2,3-triazol-4-yl)methanone(**3r**). Yield: 28 mg (58 %); white solid, m.p. 158 ^oC; ^1H NMR (400 MHz, DMSO-d_6): δ 8.78 (s, 1H), 8.45 (s, 1H), 8.21 (d, *J* = 8.4 Hz, 1H), 7.87 (d, *J* = 8.4 Hz, 1H); ^{13}C NMR (100 MHz, DMSO-d_6): δ 183.4, 145.9, 137.1, 136.6, 132.2, 131.9, 131.4, 130.4, 128.0; ESI-HRMS: Calcd for $C_9H_5Cl_2N_3O$ $[M+H]^+$ 241.9882, found 241.9882.

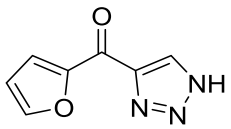

Furan-2-yl(1*H*-1,2,3-triazol-4-yl)methanone (**3s**). Yield: 26 mg (80 %); white solid, m.p. 140 ^oC; ^1H NMR (400 MHz, DMSO-d_6) δ 8.73 (s, 1H), 8.14 (s, 1H), 7.99 (s, 1H), 6.81 (d, *J* = 1.2 Hz, 1H); ^{13}C NMR (100 MHz, DMSO-d_6) δ 172.4, 151.1, 149.2, 145.3, 122.4, 113.3; ESI-HRMS: Calcd for $C_7H_5N_3O_2$ $[M+H]^+$164.0455, found 164.0455.

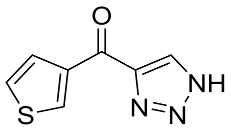

Thiophen-3-yl(1*H*-1,2,3-triazol-4-yl)methanone (**3t**). Yield: 24 mg (66 %); yellow solid, m.p. 131 ^oC; ^1H NMR (400 MHz, $CDCl_3$): δ 8.93 (s, 1H), 8.44 (s, 1H), 7.91 (s, 1H), 7.39 (s, 1H); ^{13}C NMR (100 MHz, $CDCl_3$): δ 179.2, 146.7, 140.6, 136.7, 128.3, 127.5; ESI-HRMS: Calcd for $C_7H_5N_3OS$ $[M+H]^+$ 180.0226, found 180.0226.

References:

1) F. M. A. A. EL-Taweel and M. H. Elnagdi, *J. Heterocyclic Chem.*, 2001, **38**, 981.

2) E. Ilya, B. Vasiliy, B. Nikolai, B. Tetyana, K. Uwe, L. Johann, Z.-J. Fan, E. Oleg, S. Pavel, E. Marina and D. Wim, *Eur. J. Org. Chem.*, 2014, 3684.

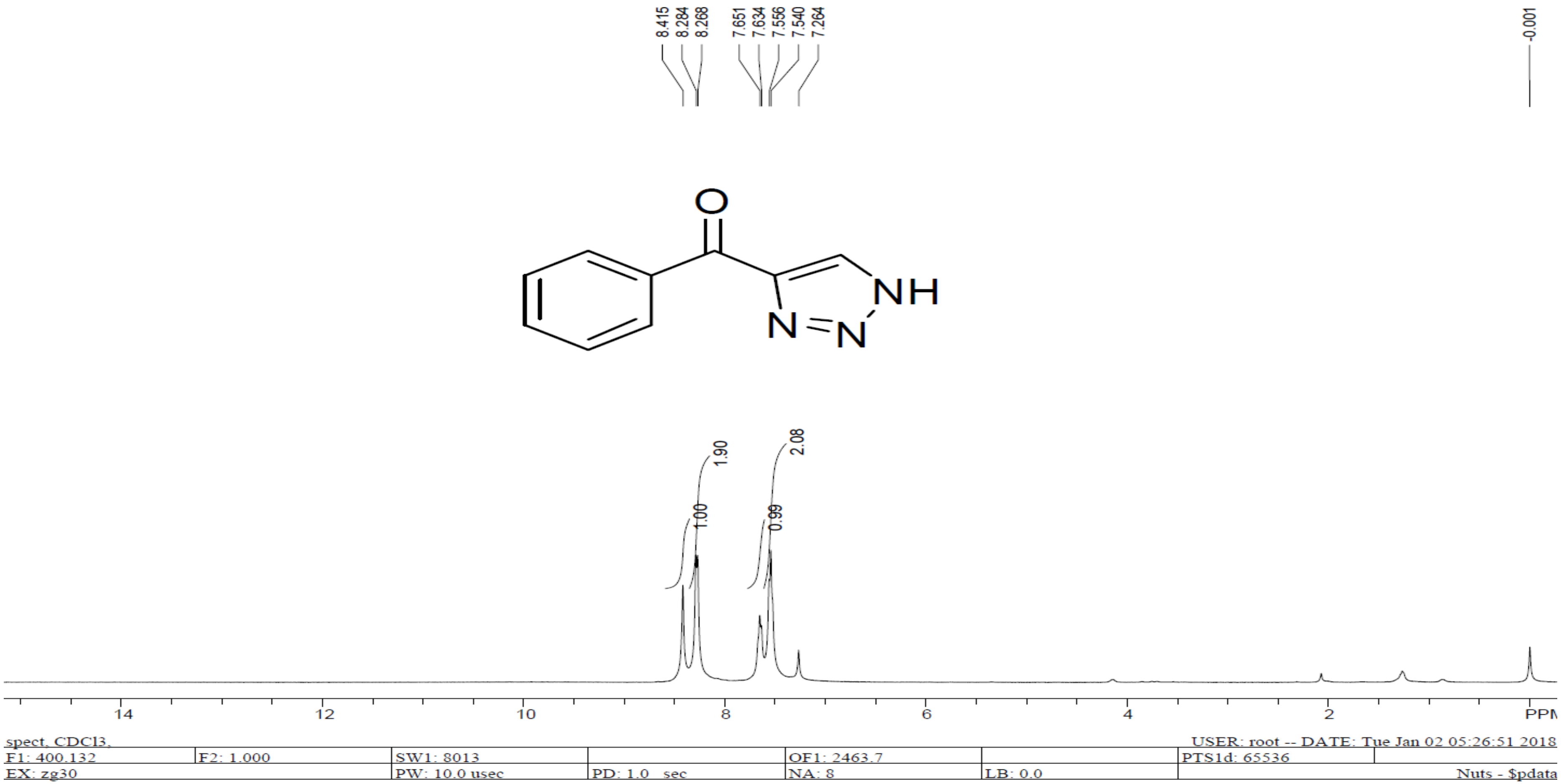

^{1}H NMR spectrum of **3a**

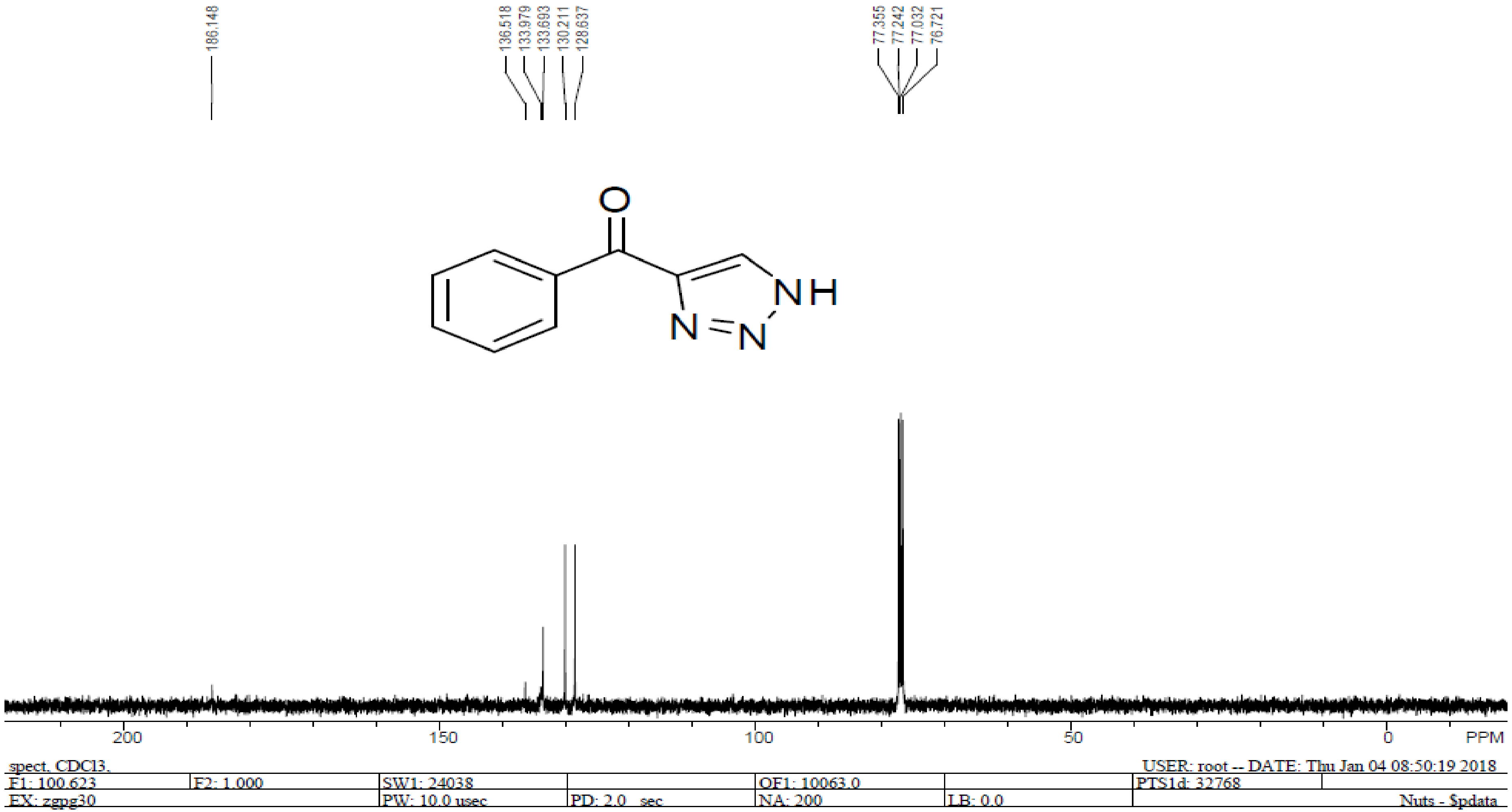

^{13}C NMR spectrum of **3b**

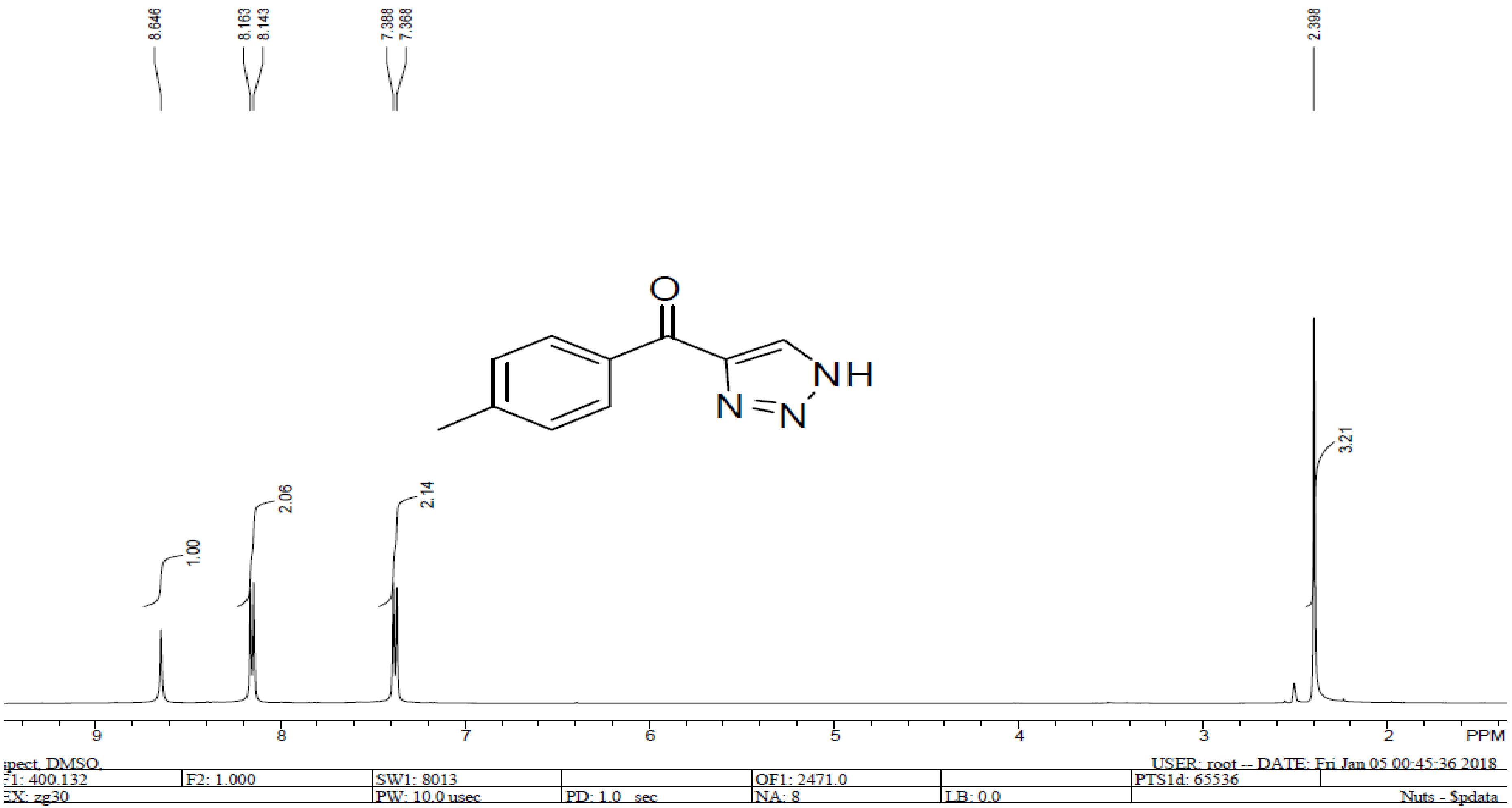

^{1}H NMR spectrum of **3b**

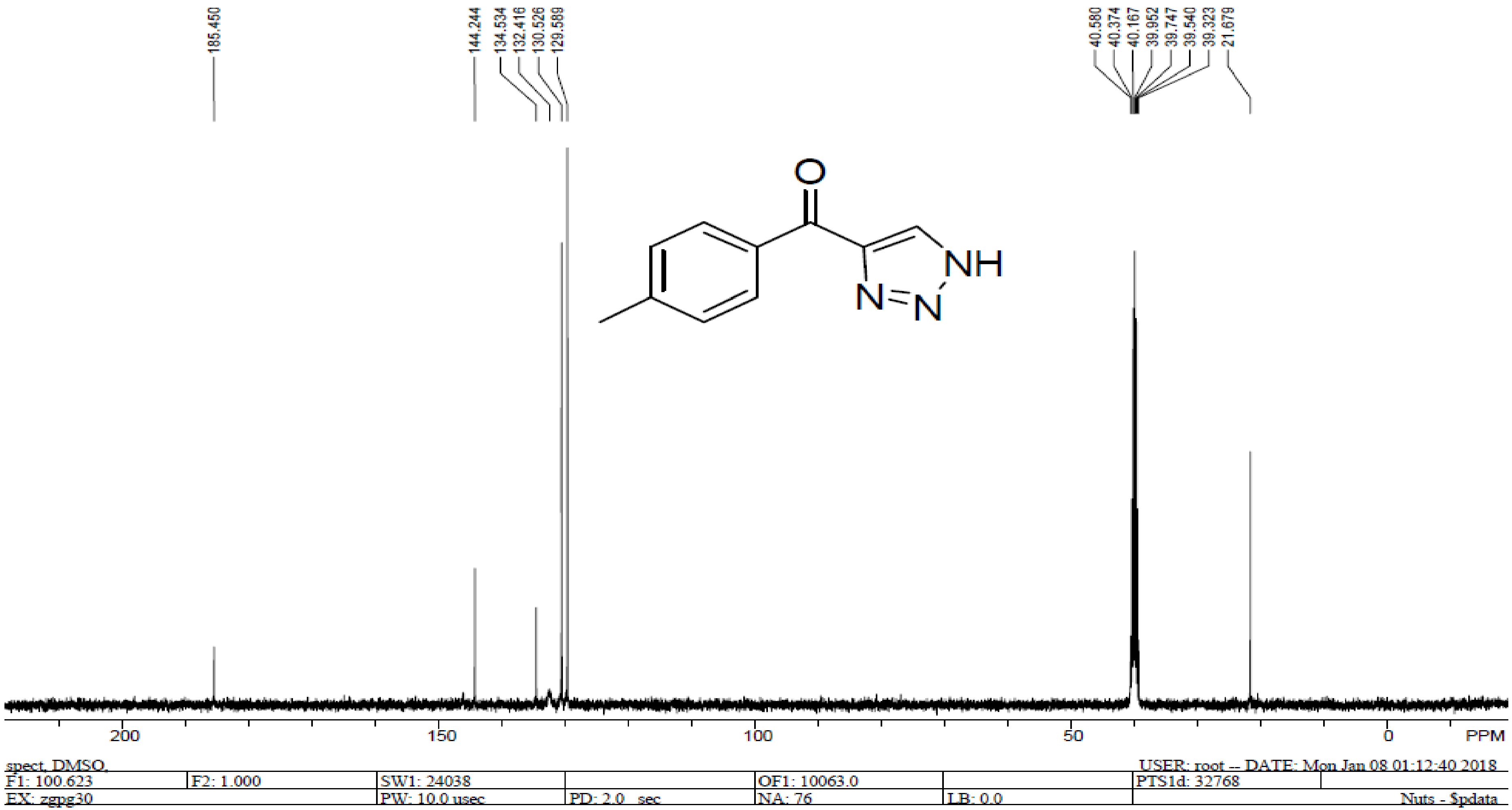

^{13}C NMR spectrum of **3b**

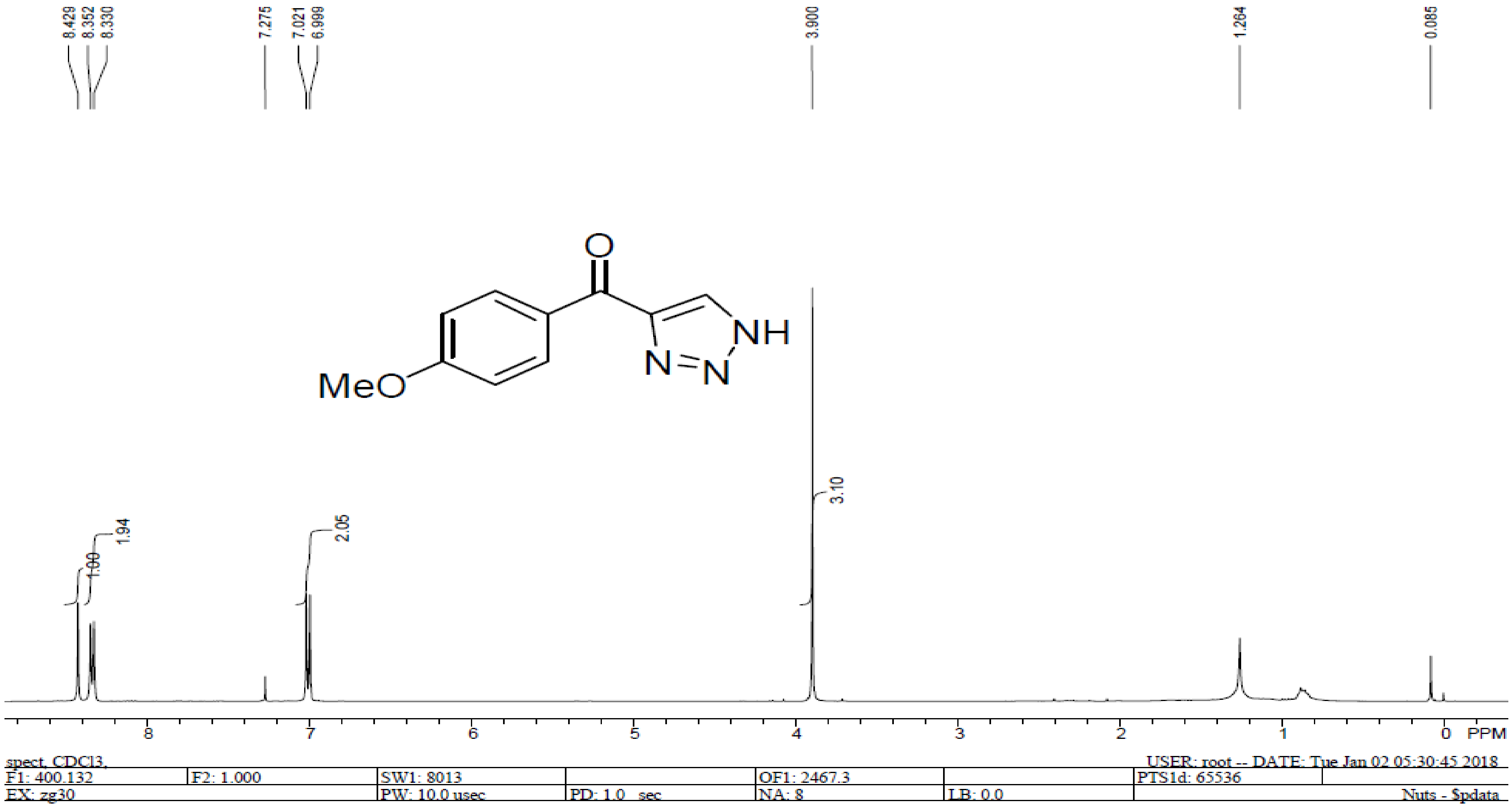

^{1}H NMR spectrum of **3c**

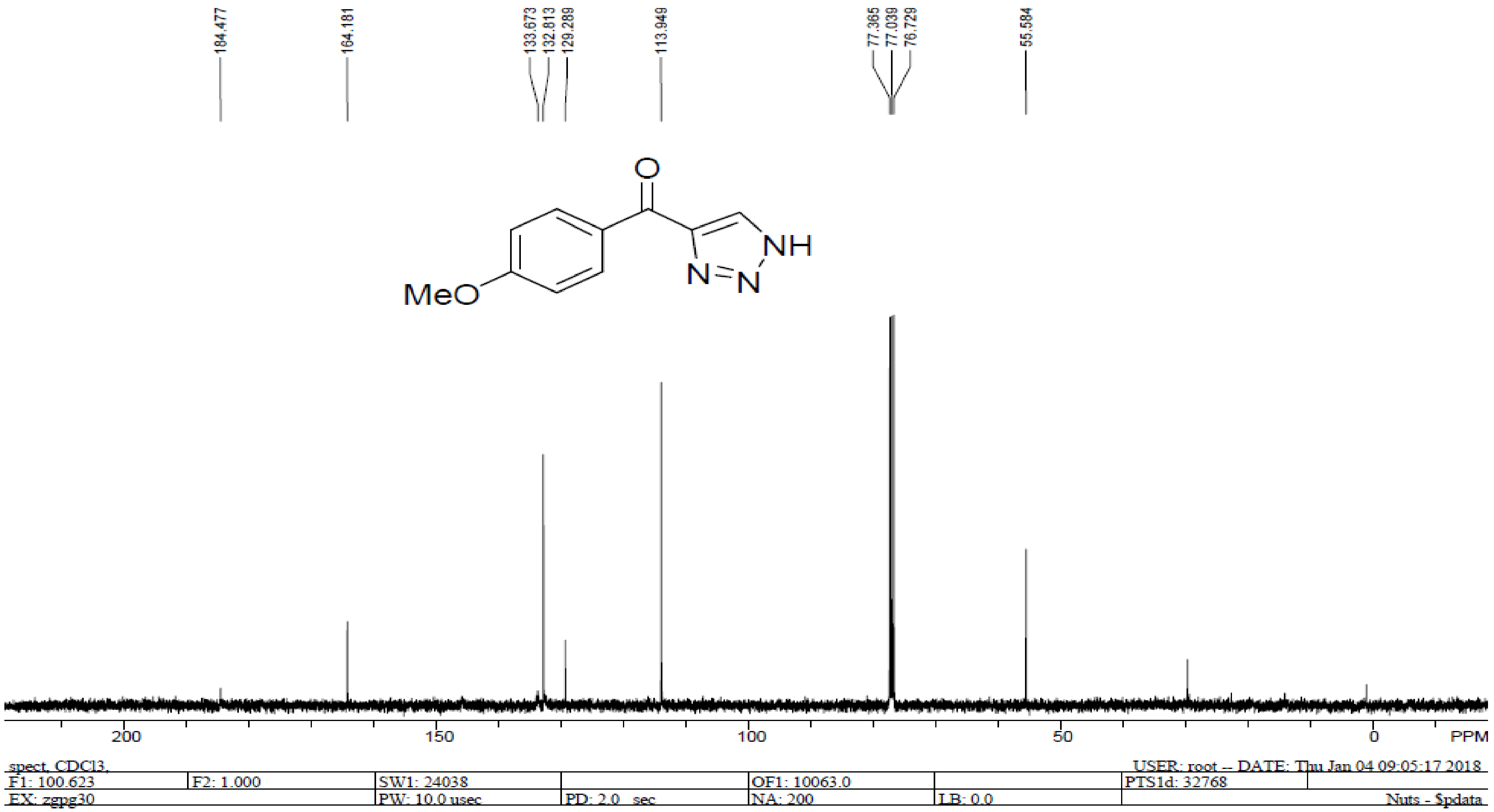

^{13}C NMR spectrum of **3c**

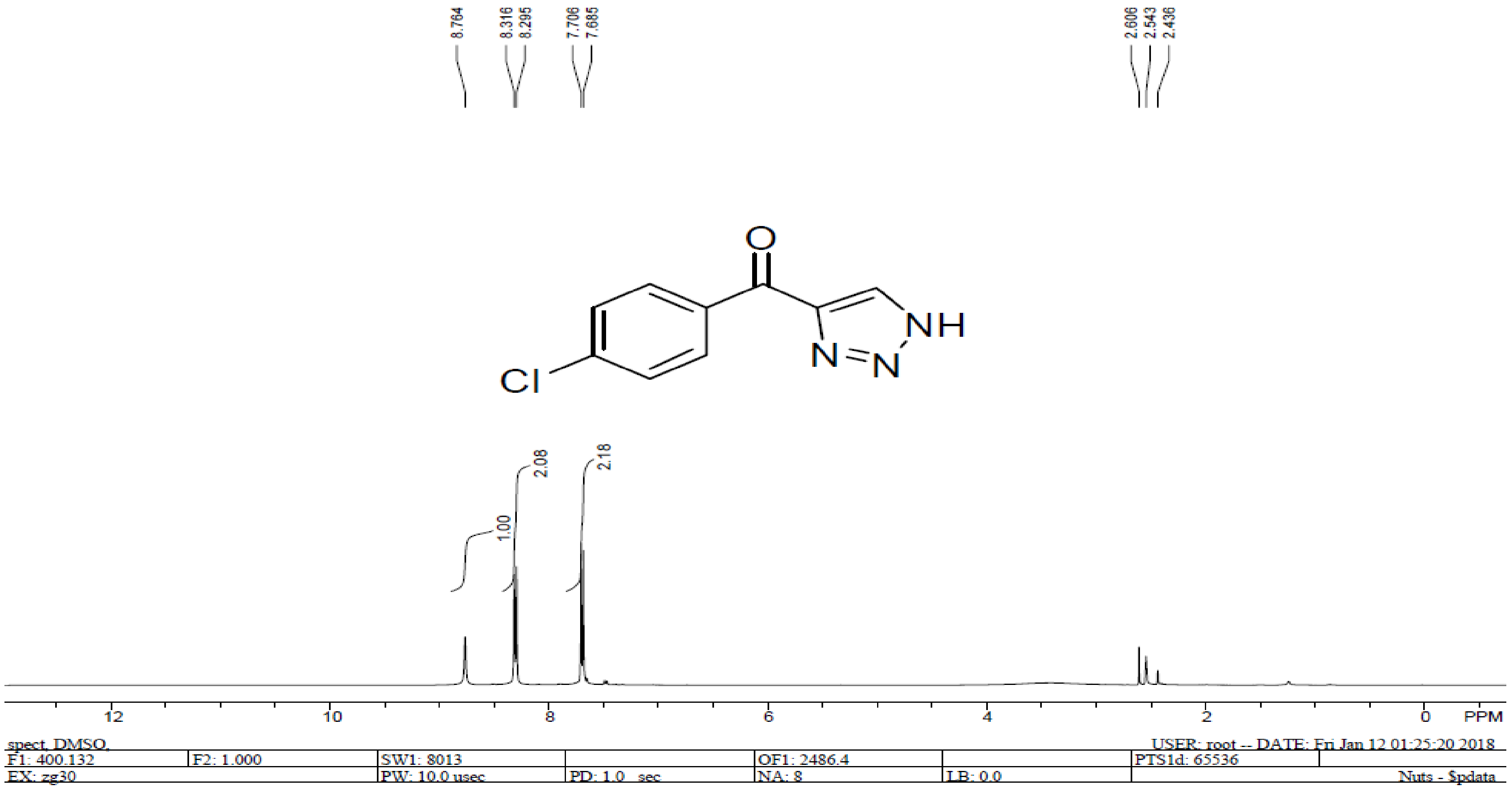

^{1}H NMR spectrum of **3d**

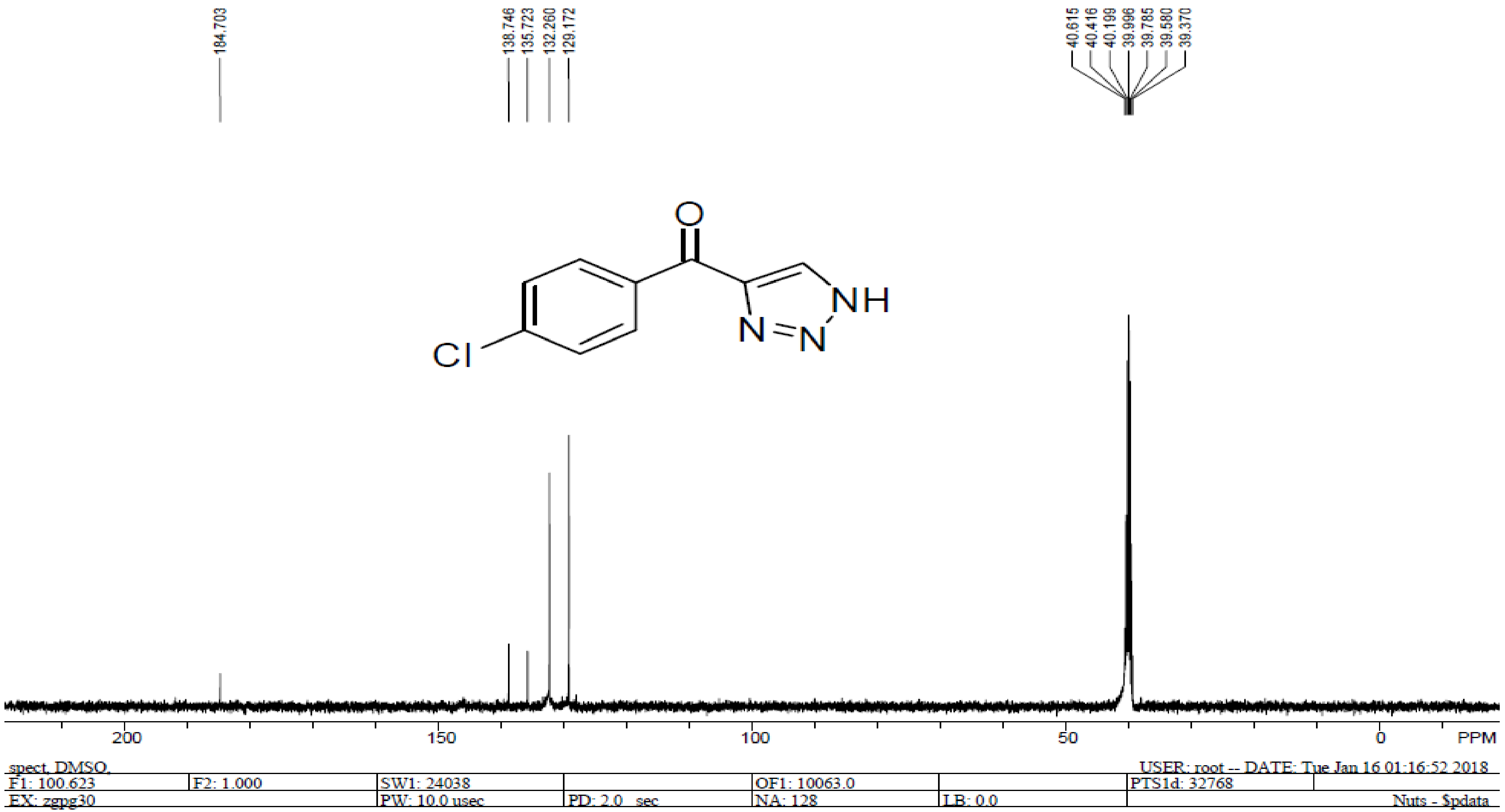

^{13}C NMR spectrum of **3d**

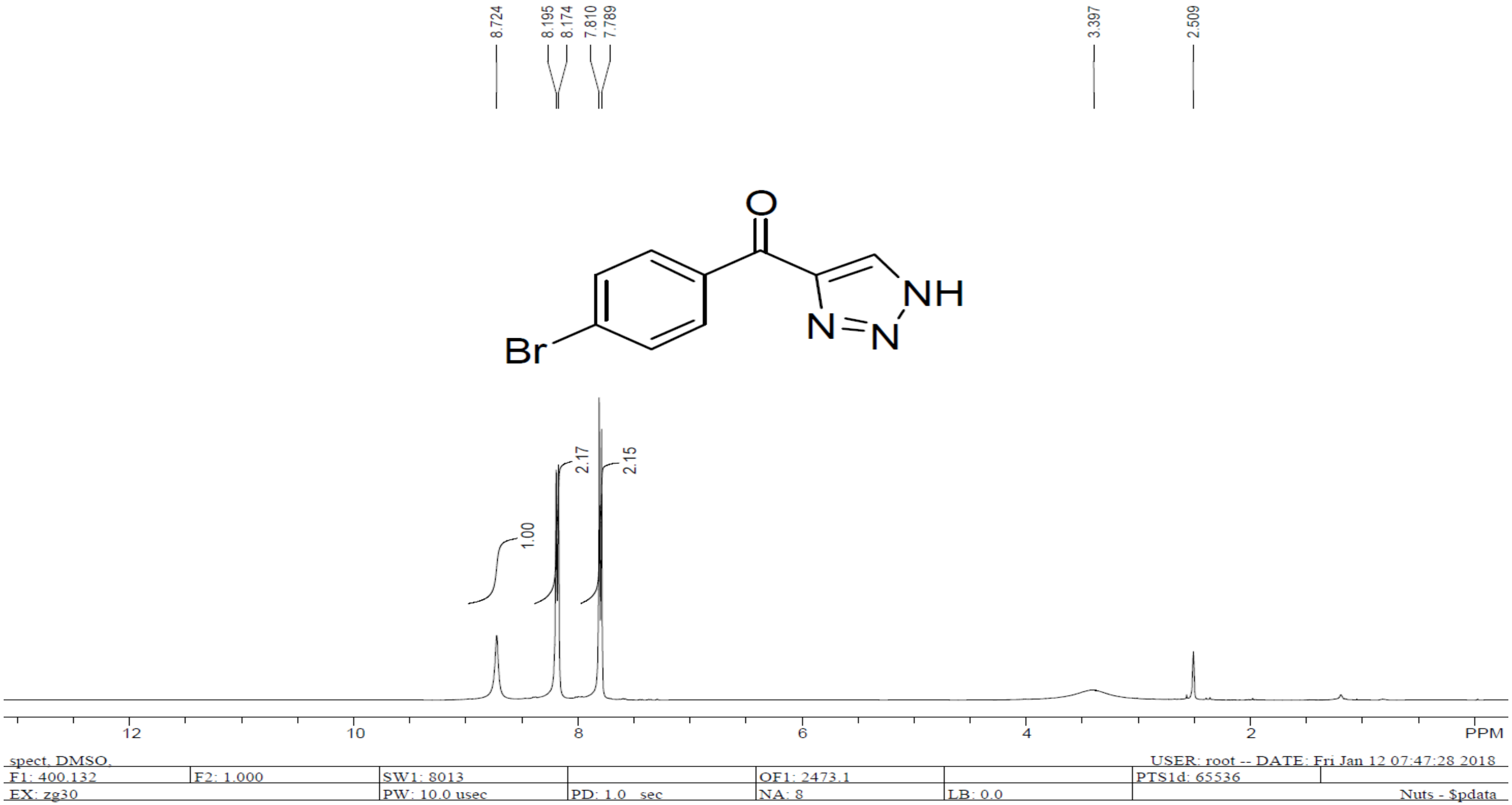

^{1}H NMR spectrum of **3e**

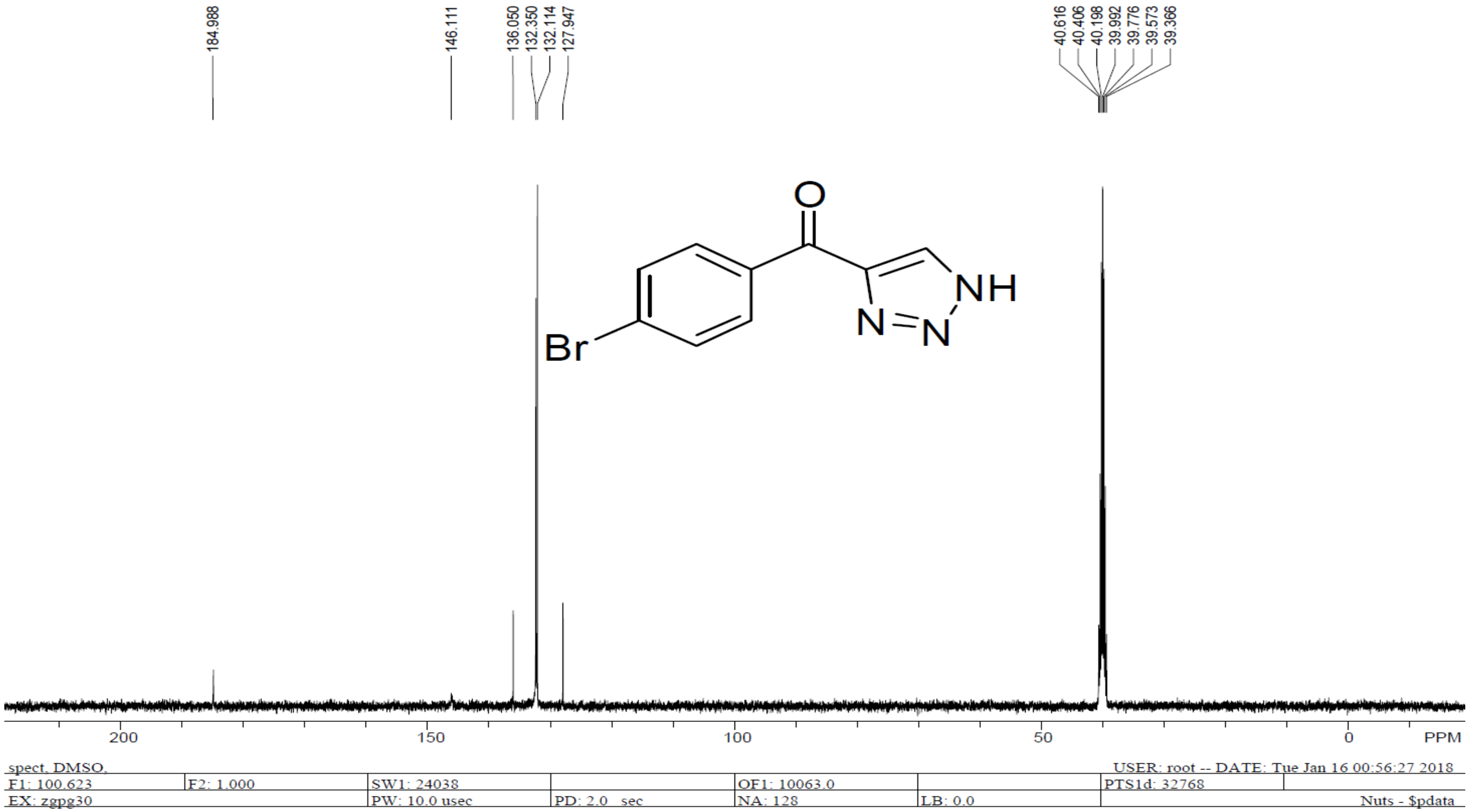

^{13}C NMR spectrum of **3e**

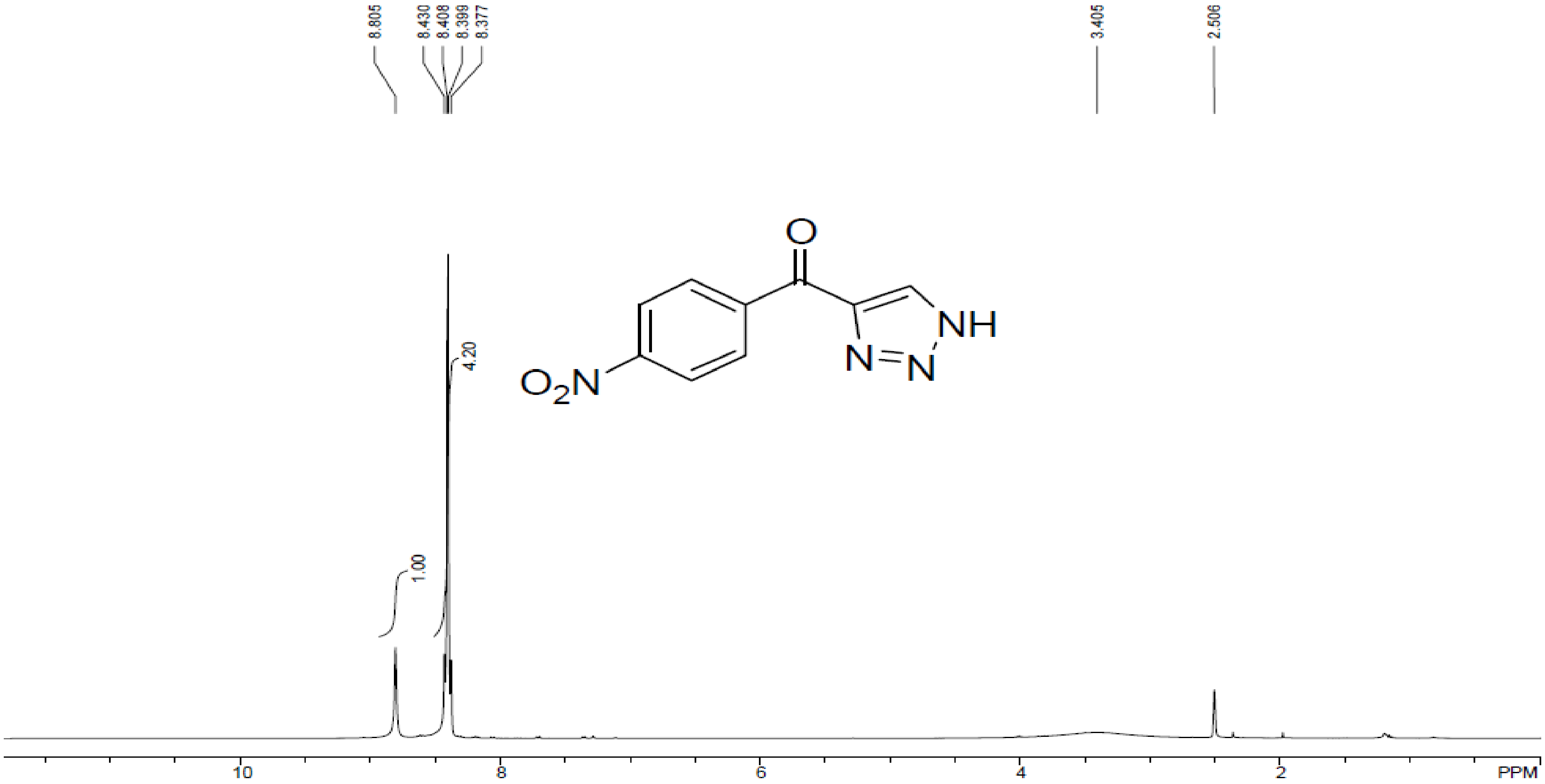

spect, DMSO,						USER: root -- DATE: Fri Jan 05 00:52:25 2018	
F1: 400.132	F2: 1.000	SW1: 8013		OF1: 2471.0		PTS1d: 65536	
EX: zg30		PW: 10.0 usec	PD: 1.0 sec	NA: 8	LB: 0.0		Nuts - $pdata

^{1}H NMR spectrum of **3f**

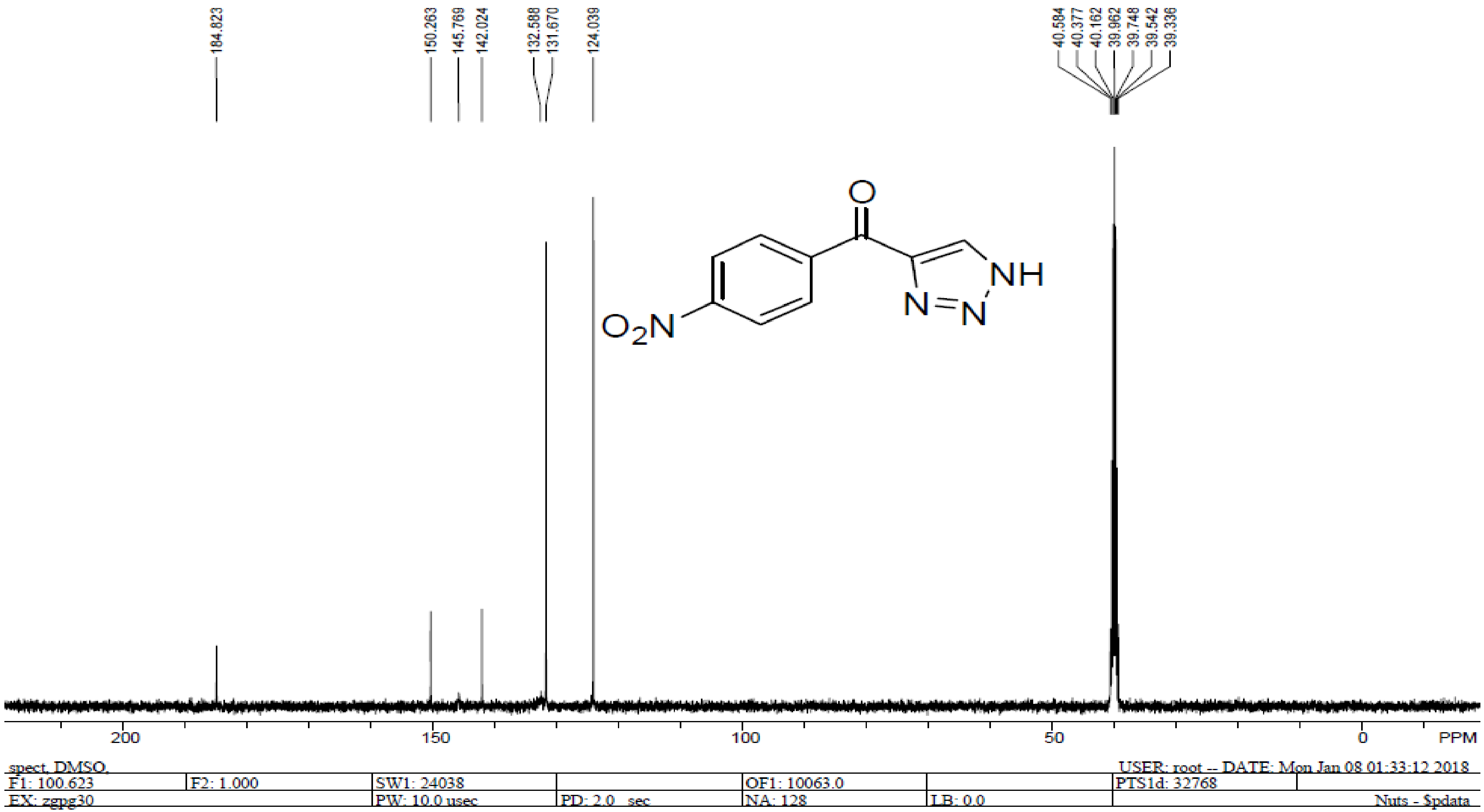

^{13}C NMR spectrum of **3f**

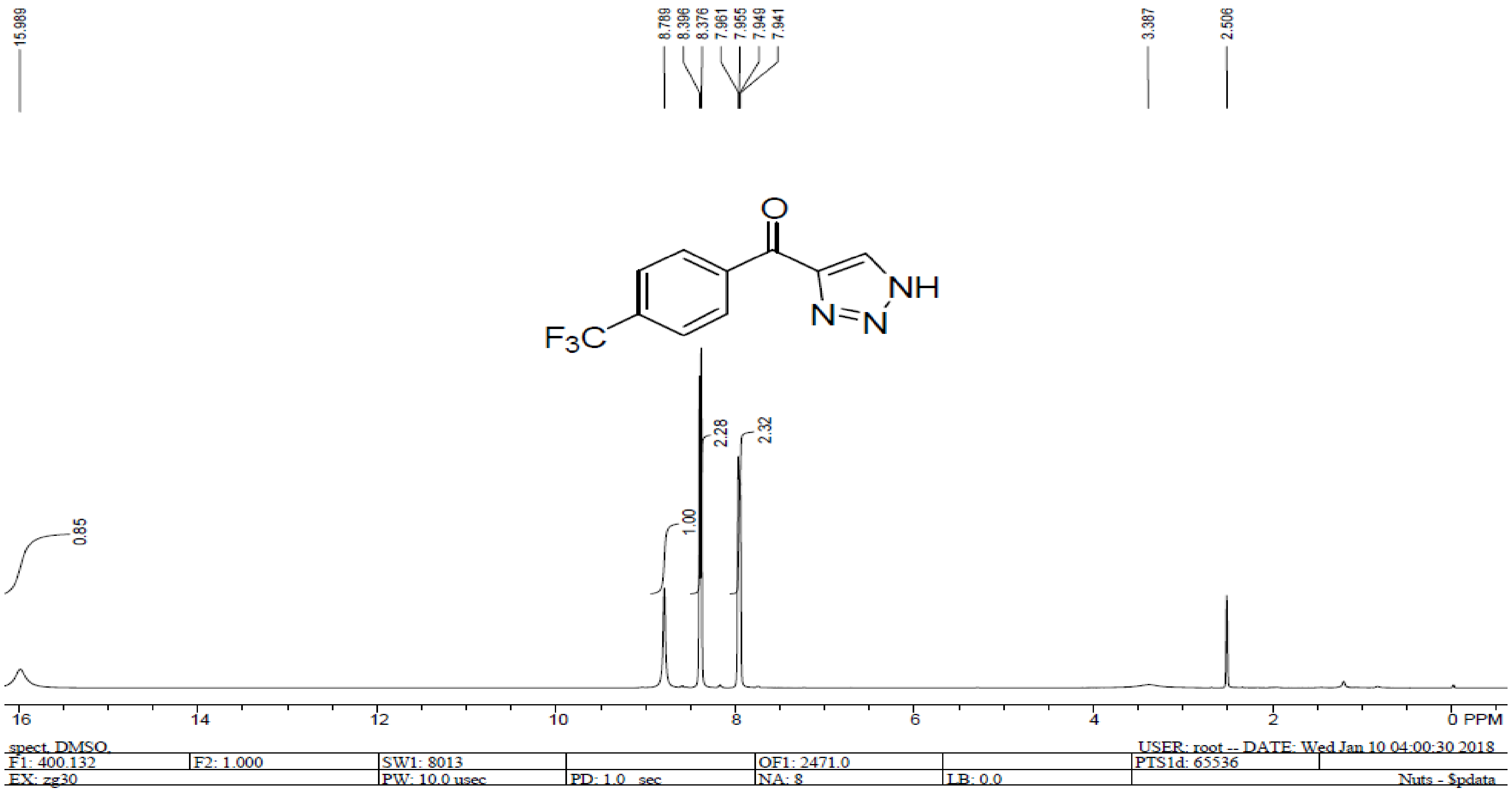

^{1}H NMR spectrum of **3g**

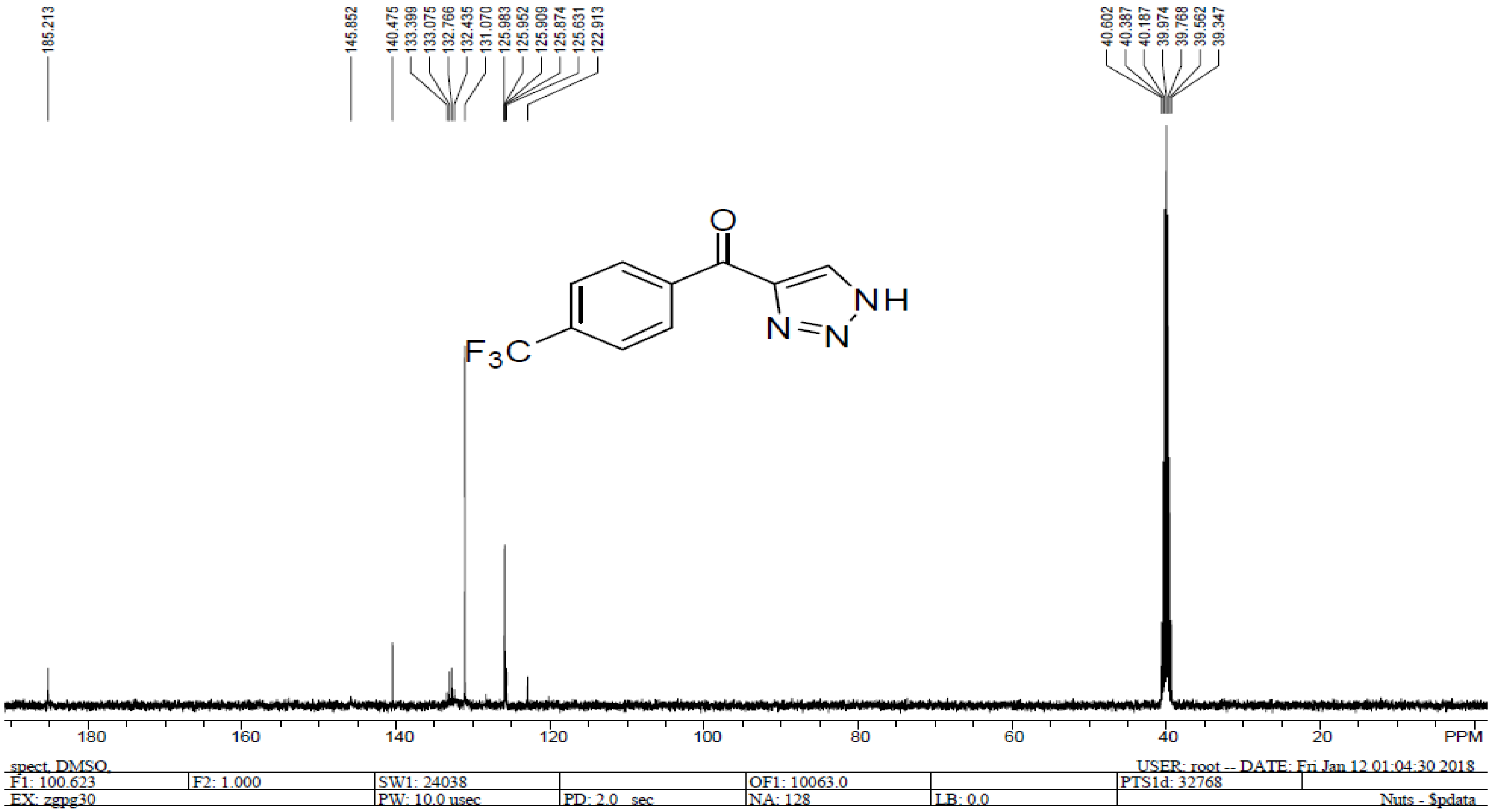

^{13}C NMR spectrum of **3g**

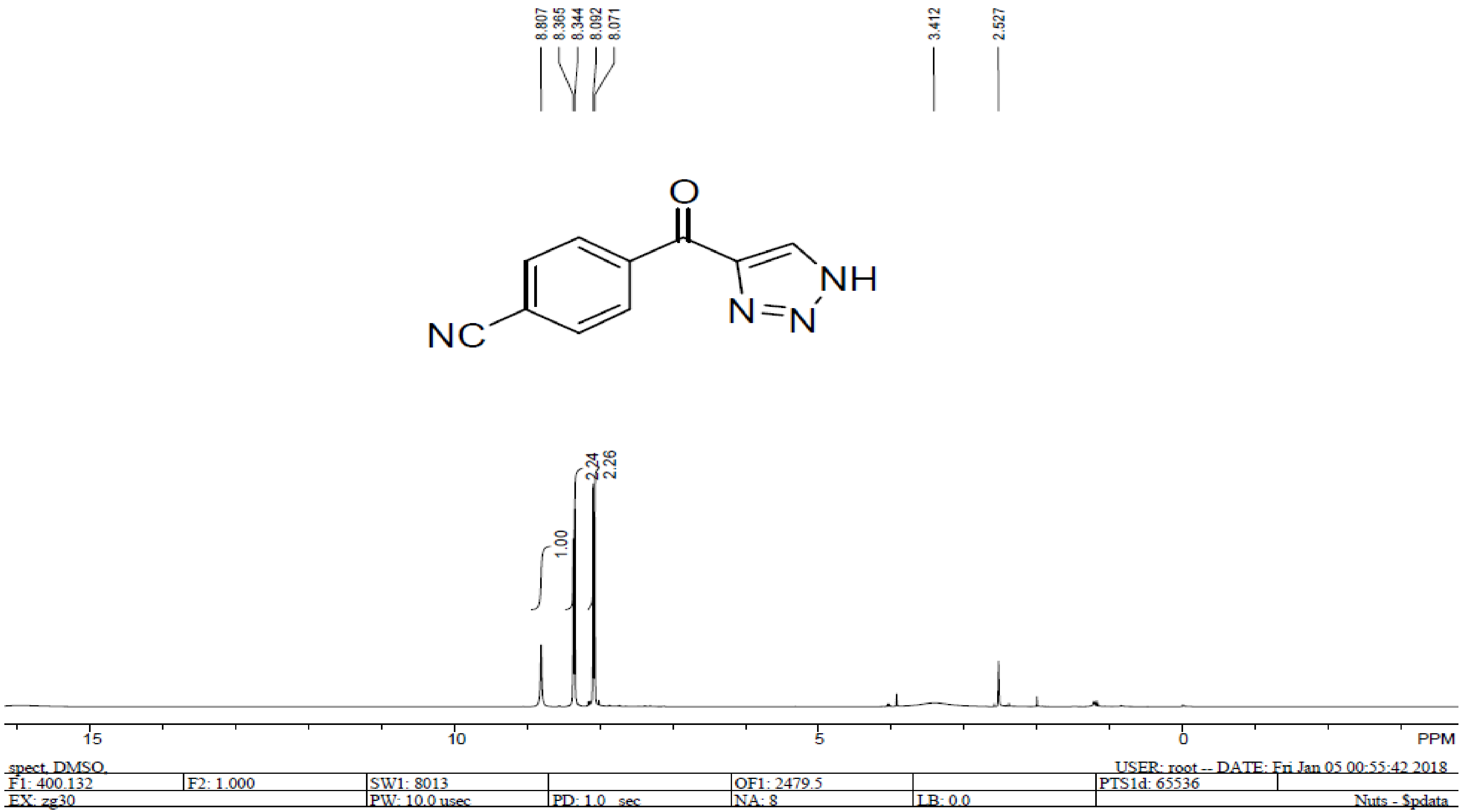

^{1}H NMR spectrum of **3h**

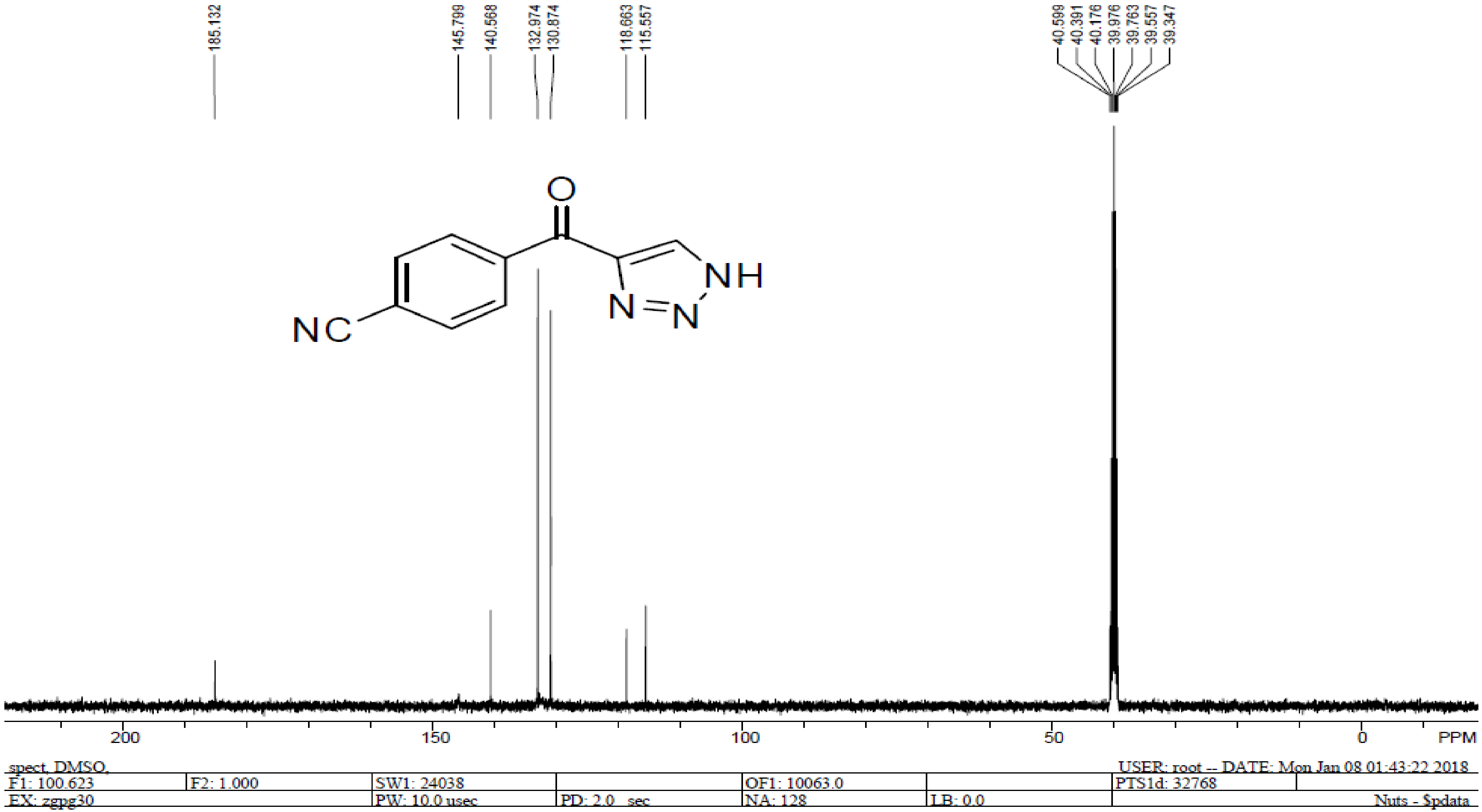

^{13}C NMR spectrum of **3h**

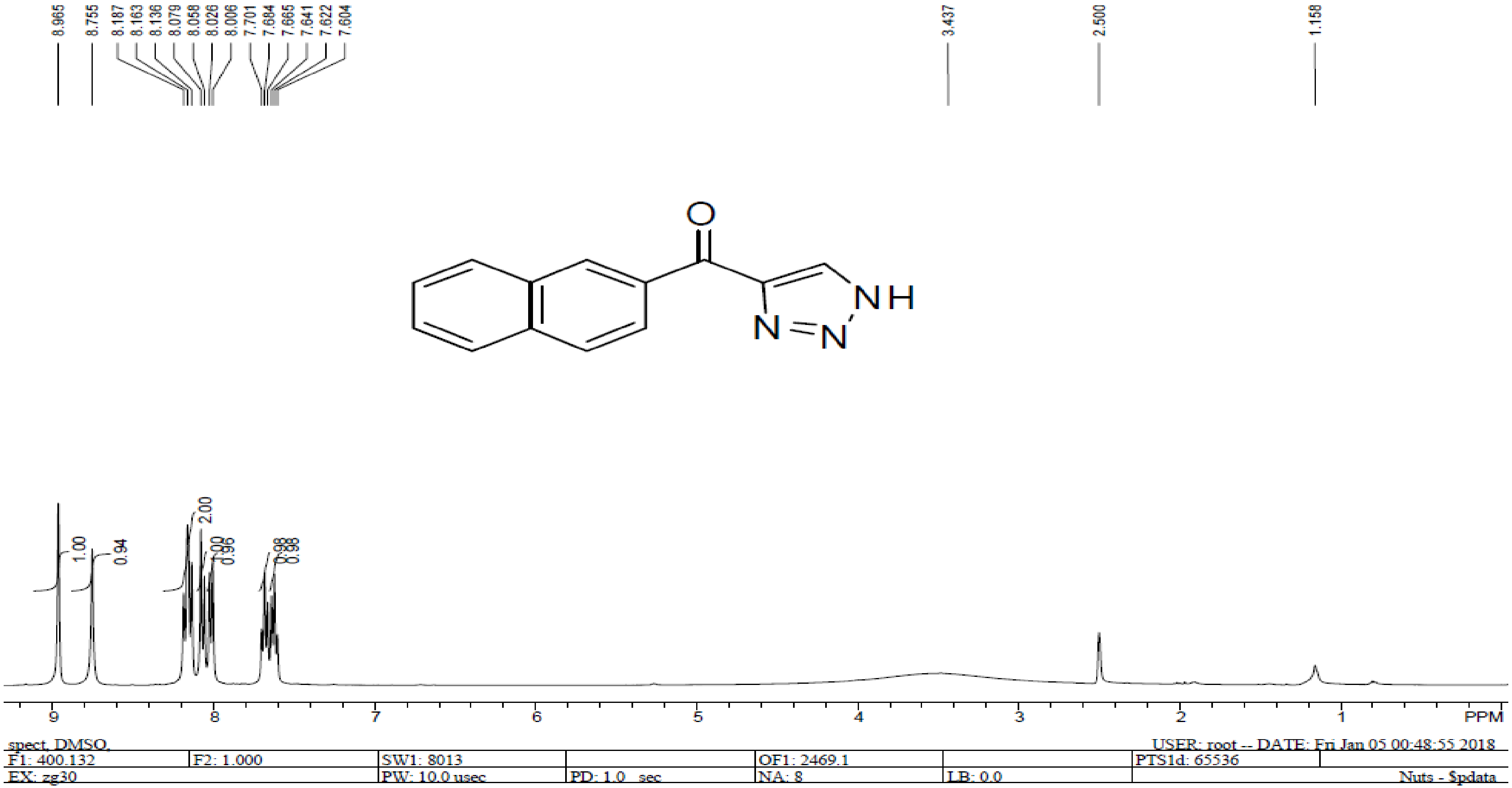

^{1}H NMR spectrum of **3i**

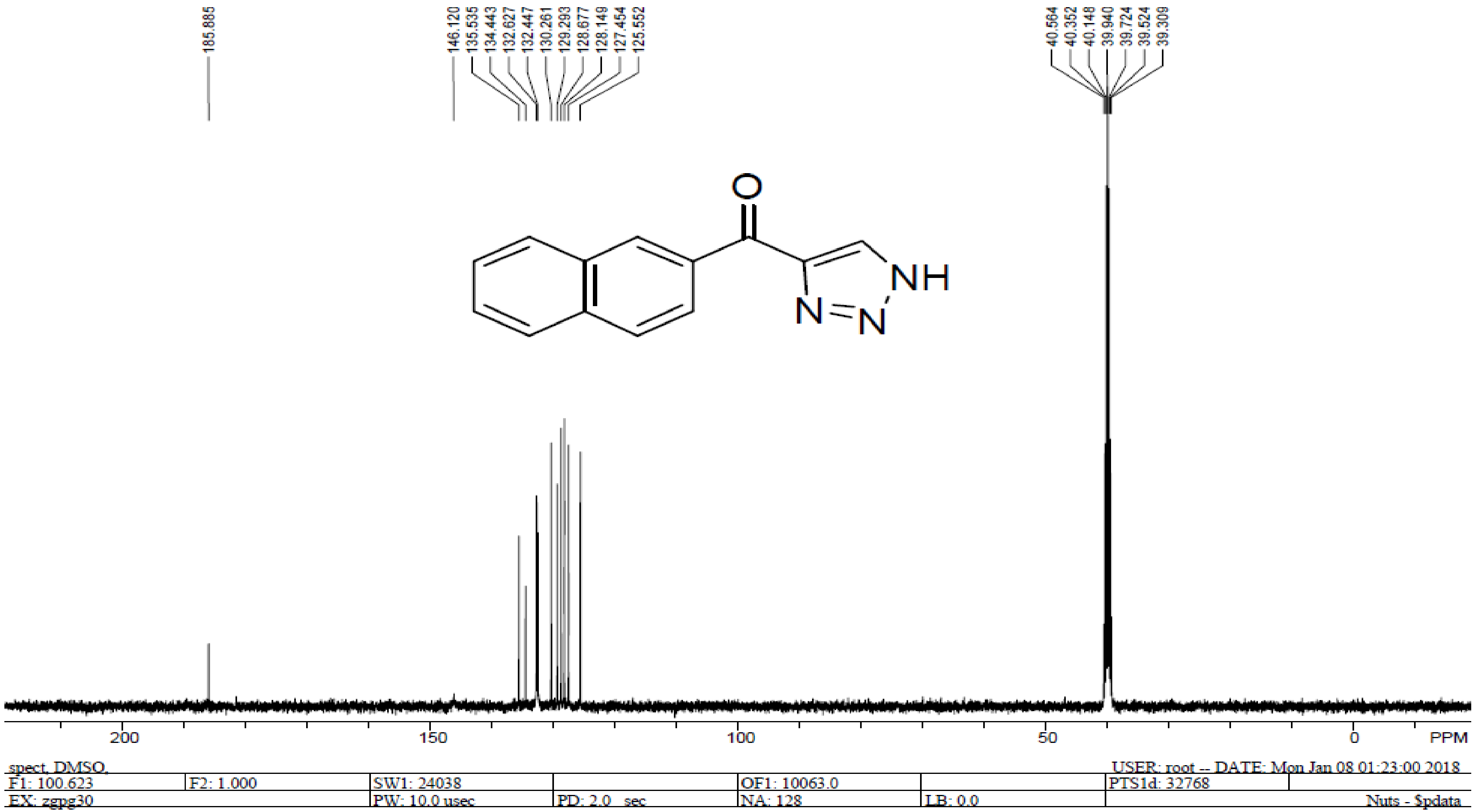

^{13}C NMR spectrum of **3i**

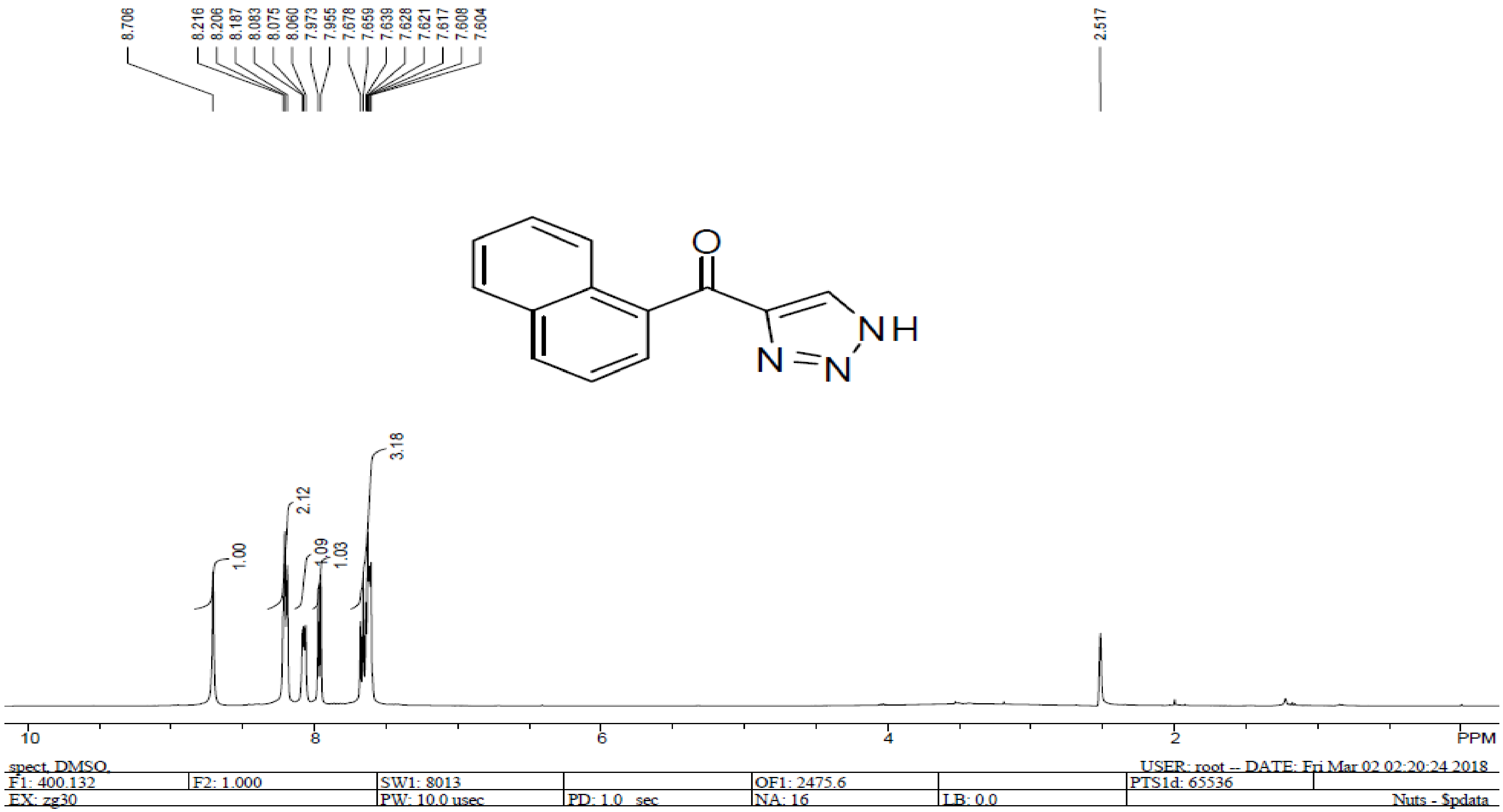

^{1}H NMR spectrum of **3j**

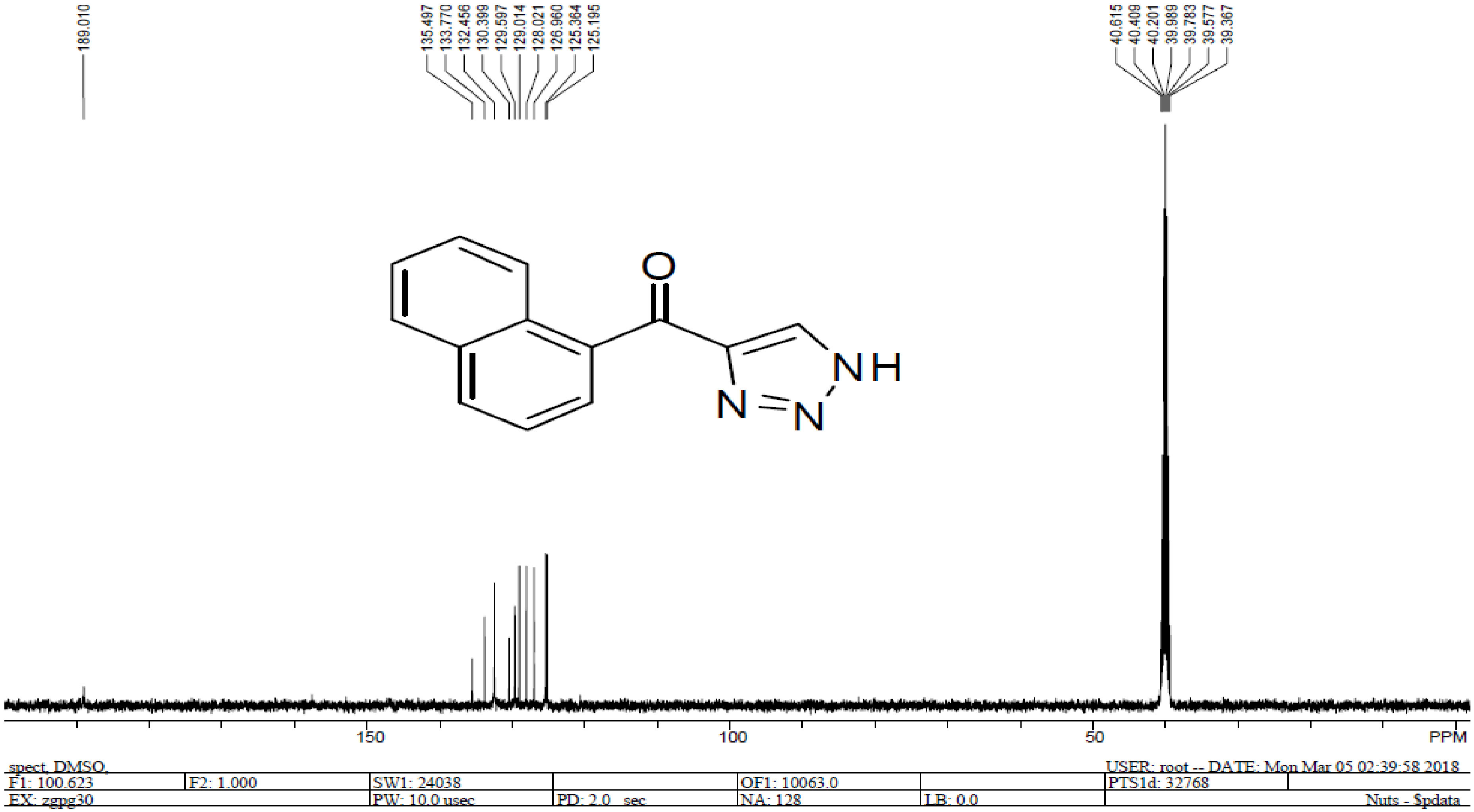

^{13}C NMR spectrum of **3j**

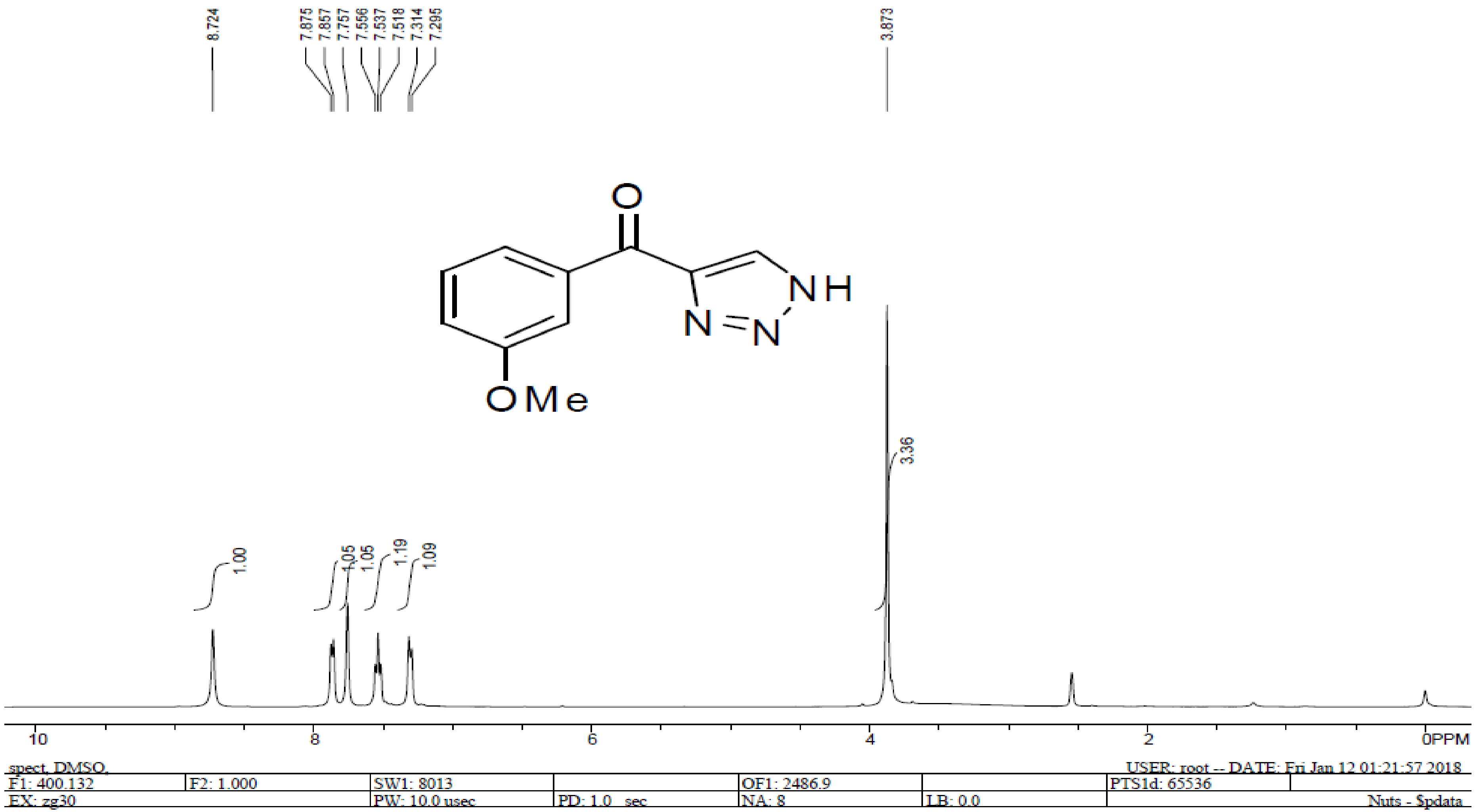

^{1}H NMR spectrum of **3k**

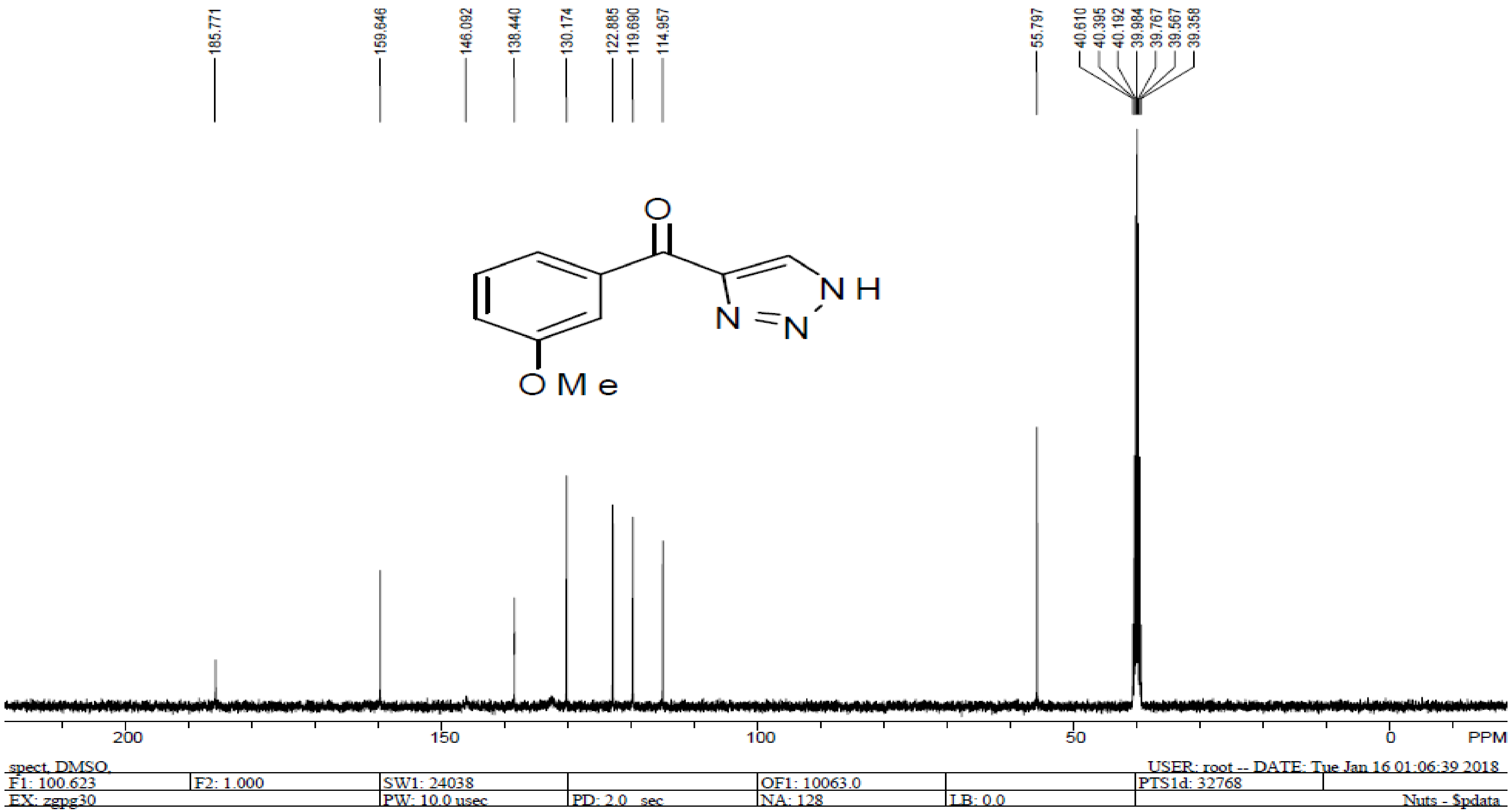

^{13}C NMR spectrum of **3k**

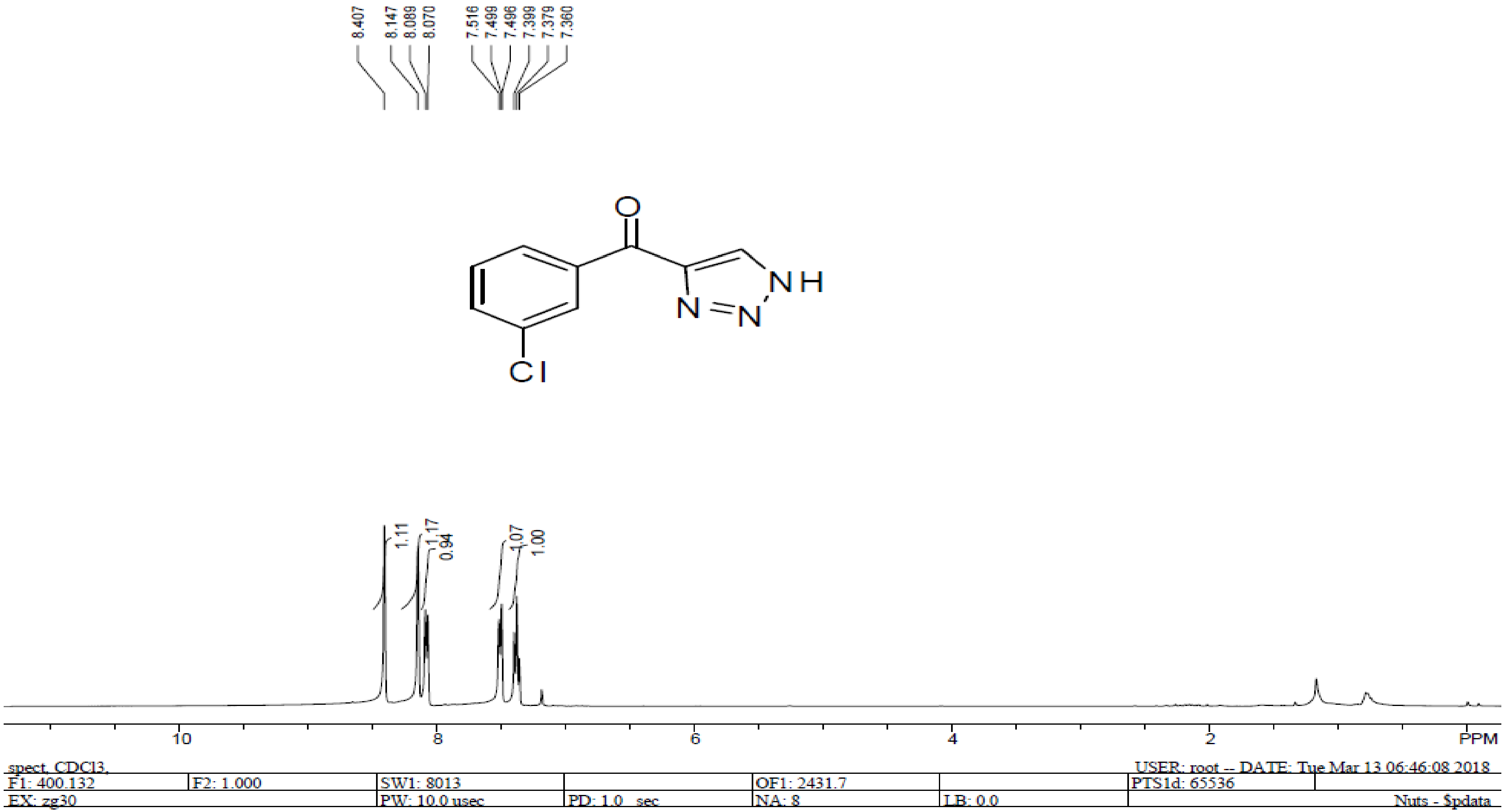

^{1}H NMR spectrum of **3l**

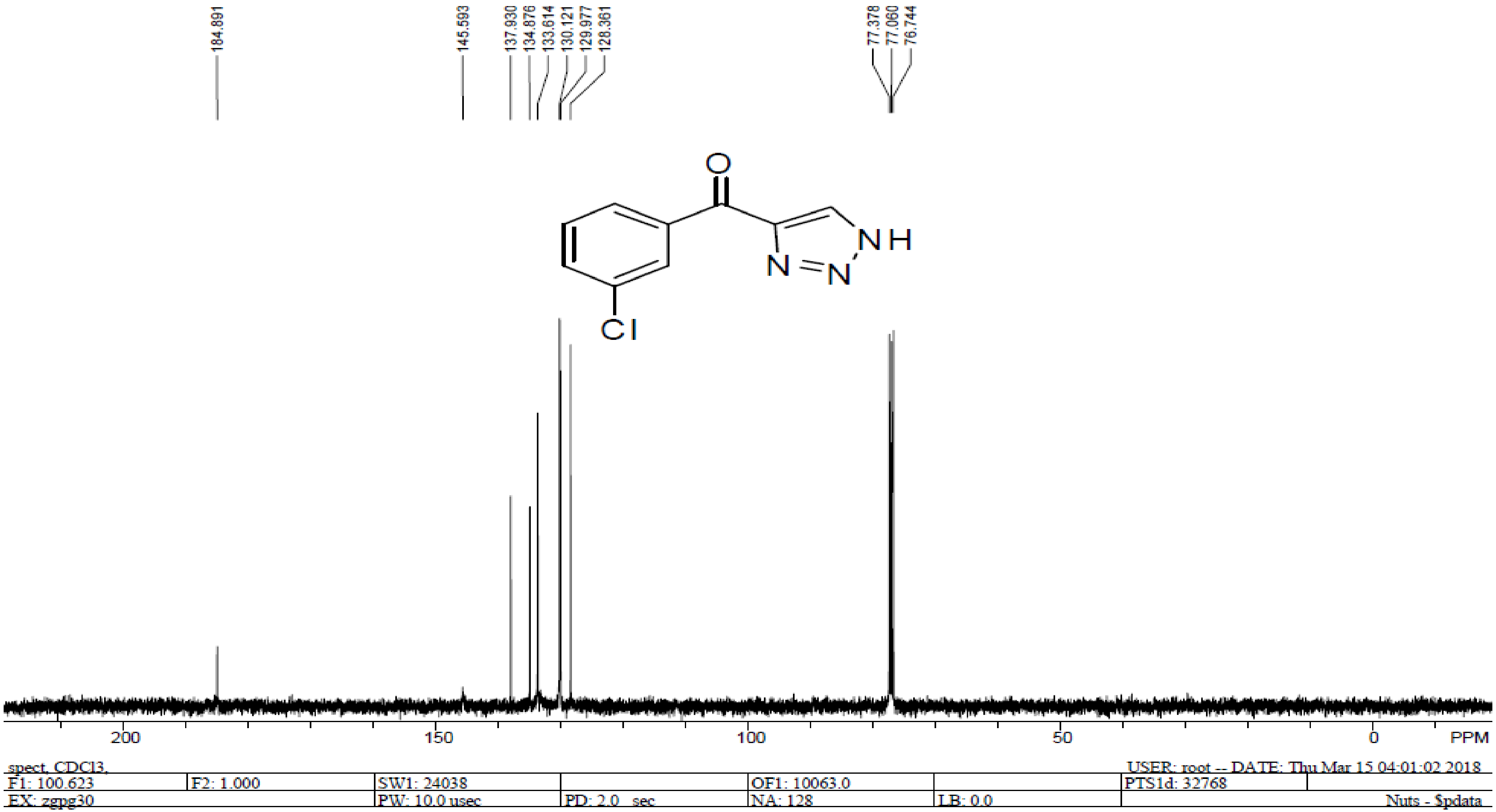

^{13}C NMR spectrum of **3l**

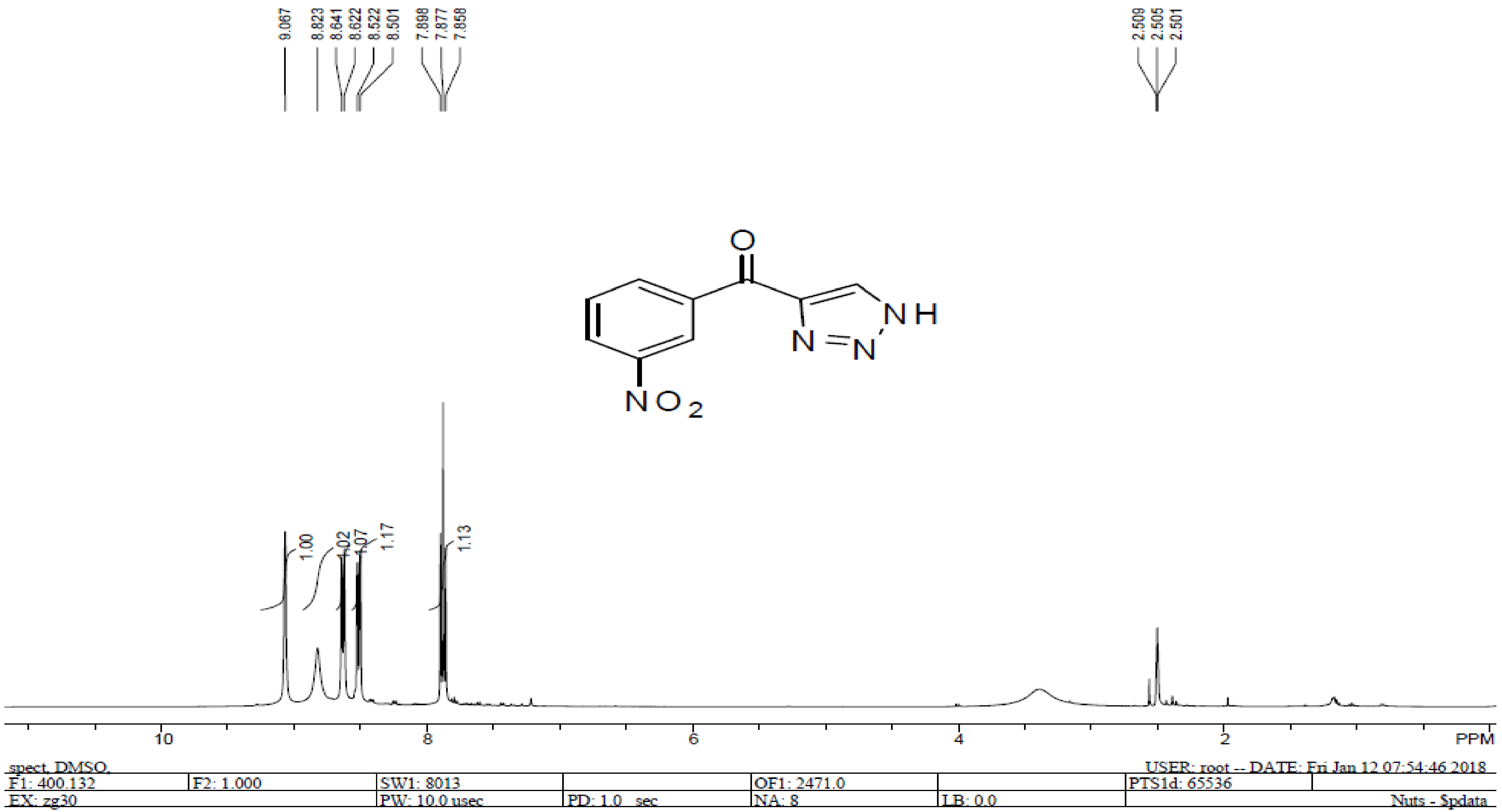

^{1}H NMR spectrum of **3m**

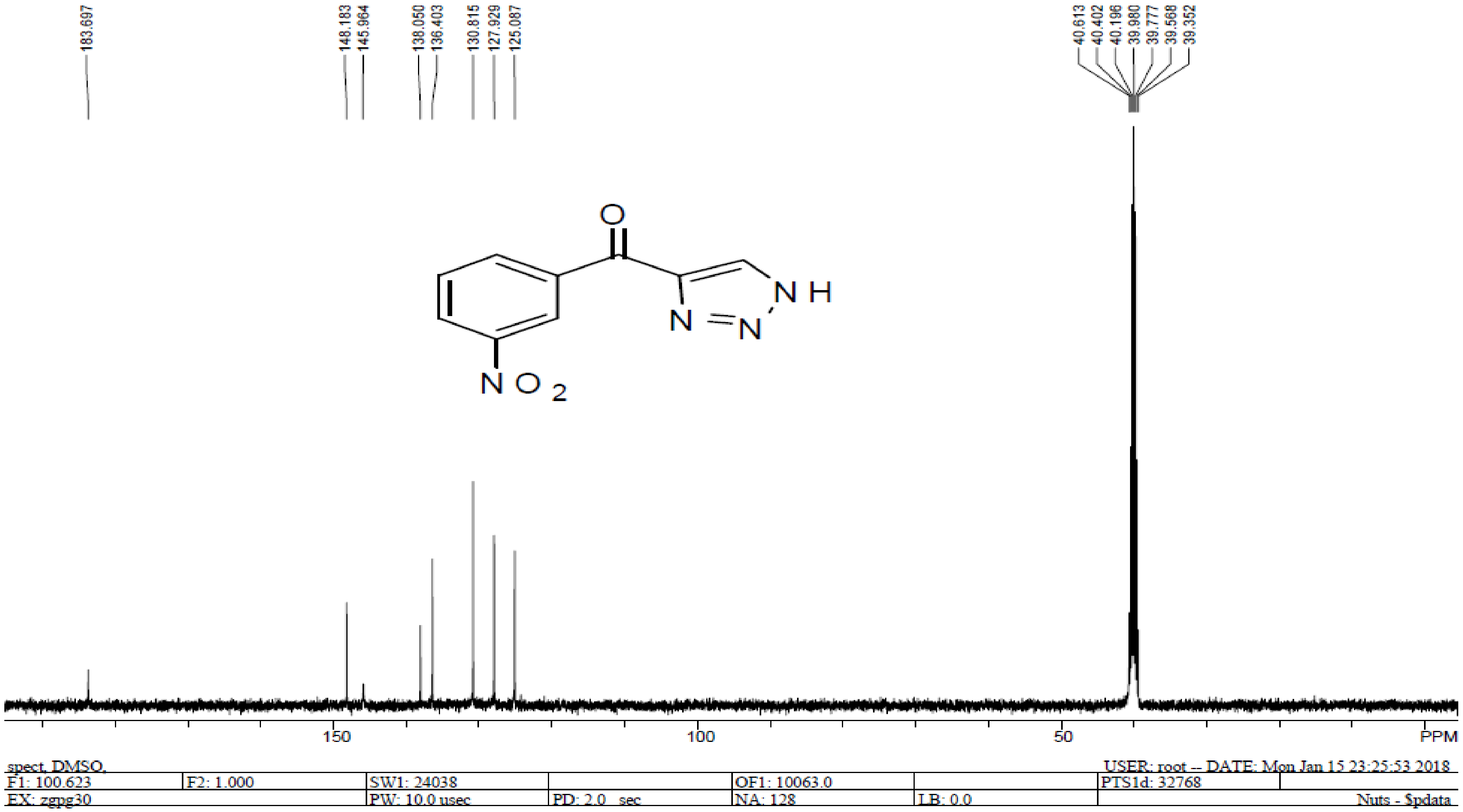

^{13}C NMR spectrum of **3m**

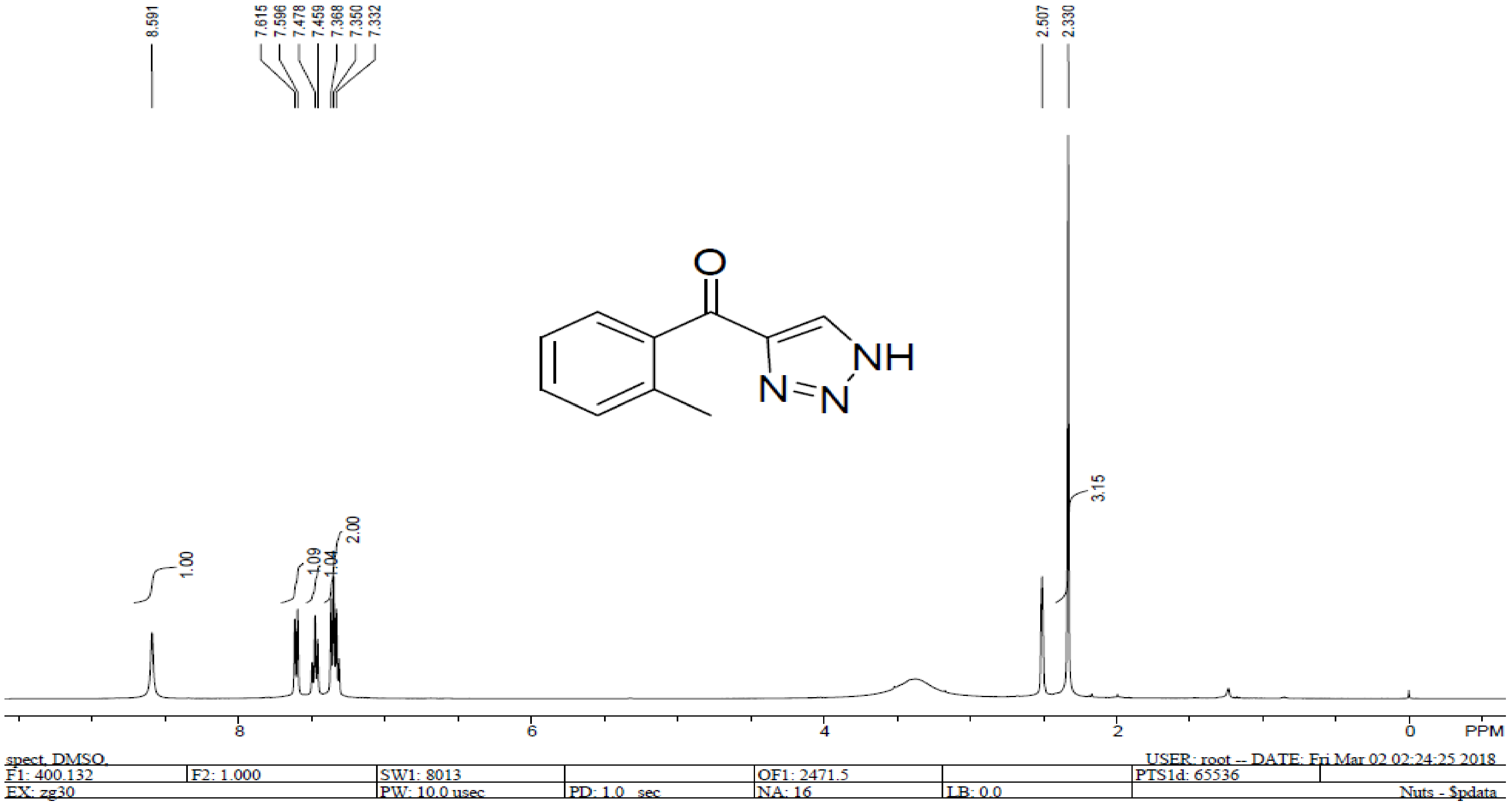

^{1}H NMR spectrum of **3n**

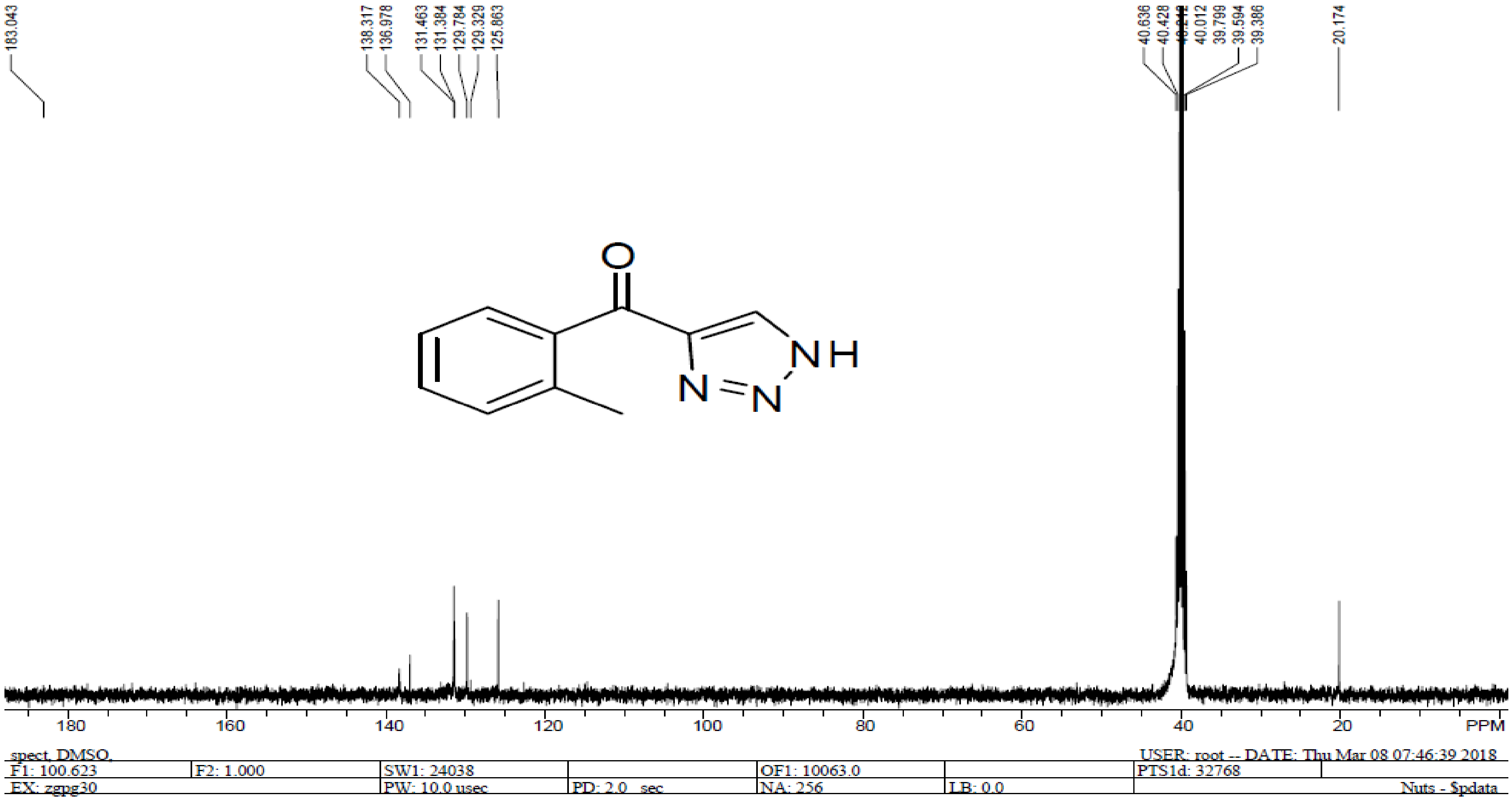

^{13}C NMR spectrum of **3n**

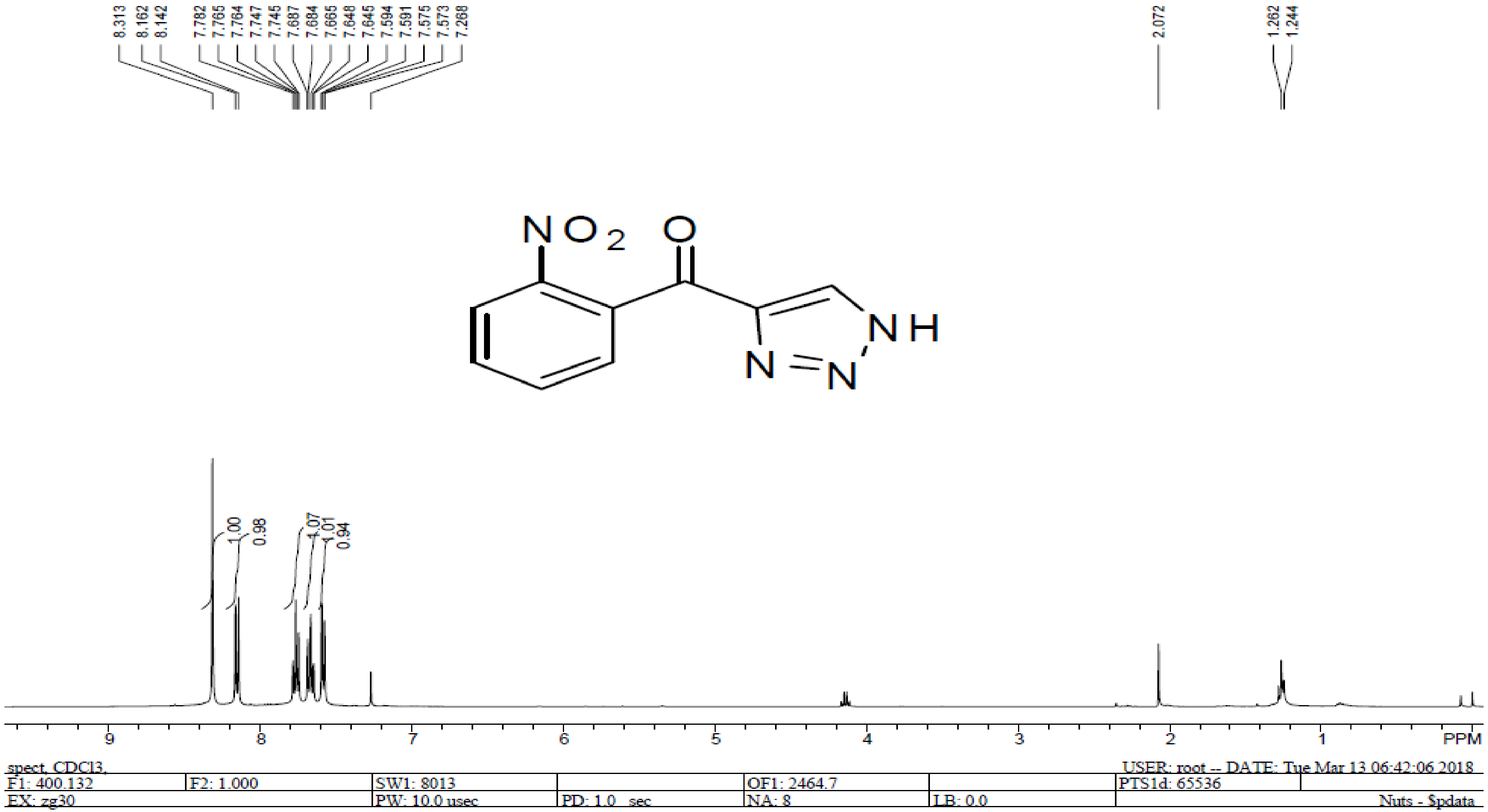

^{1}H NMR spectrum of **3o**

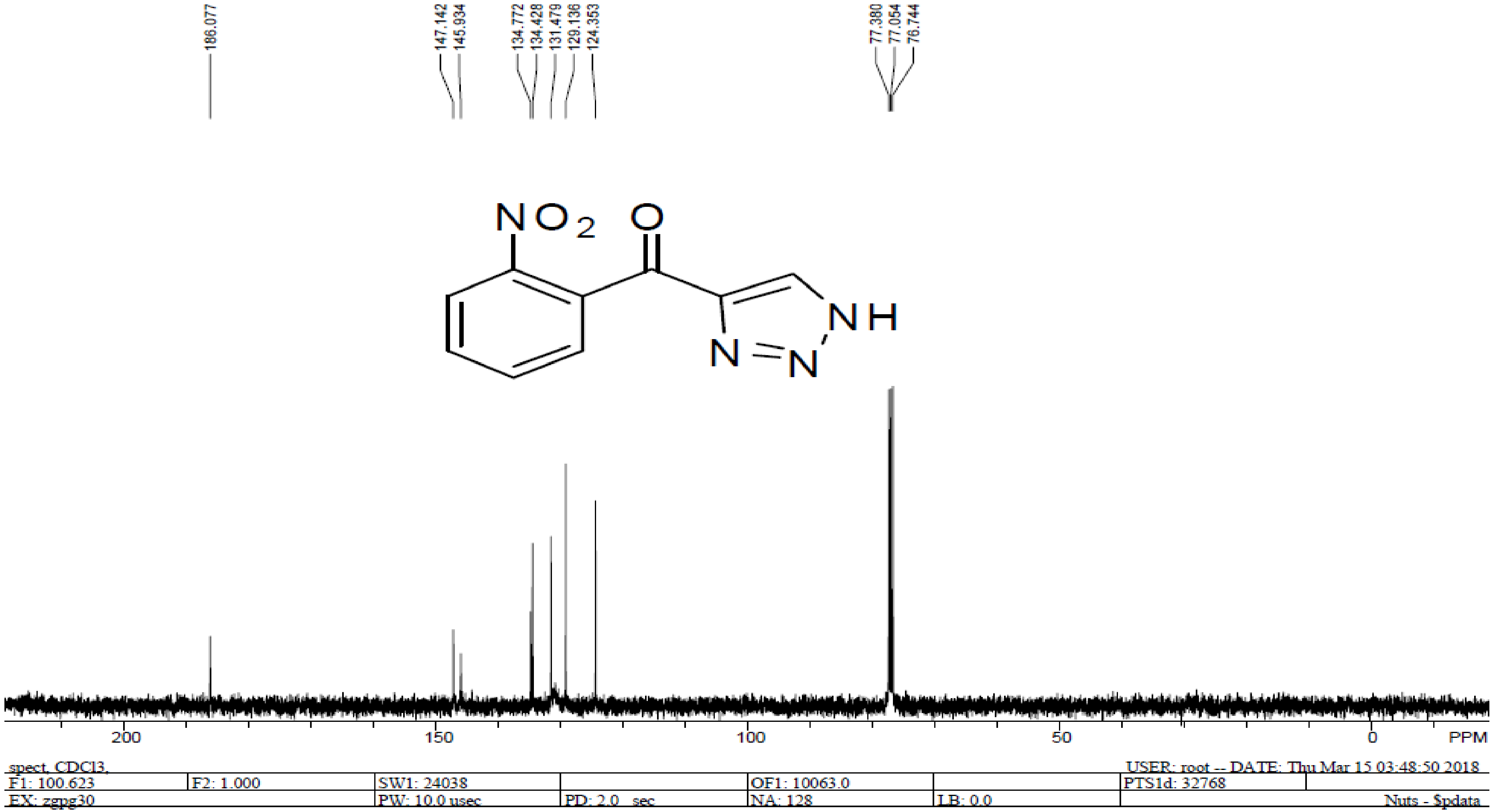

^{13}C NMR spectrum of **3o**

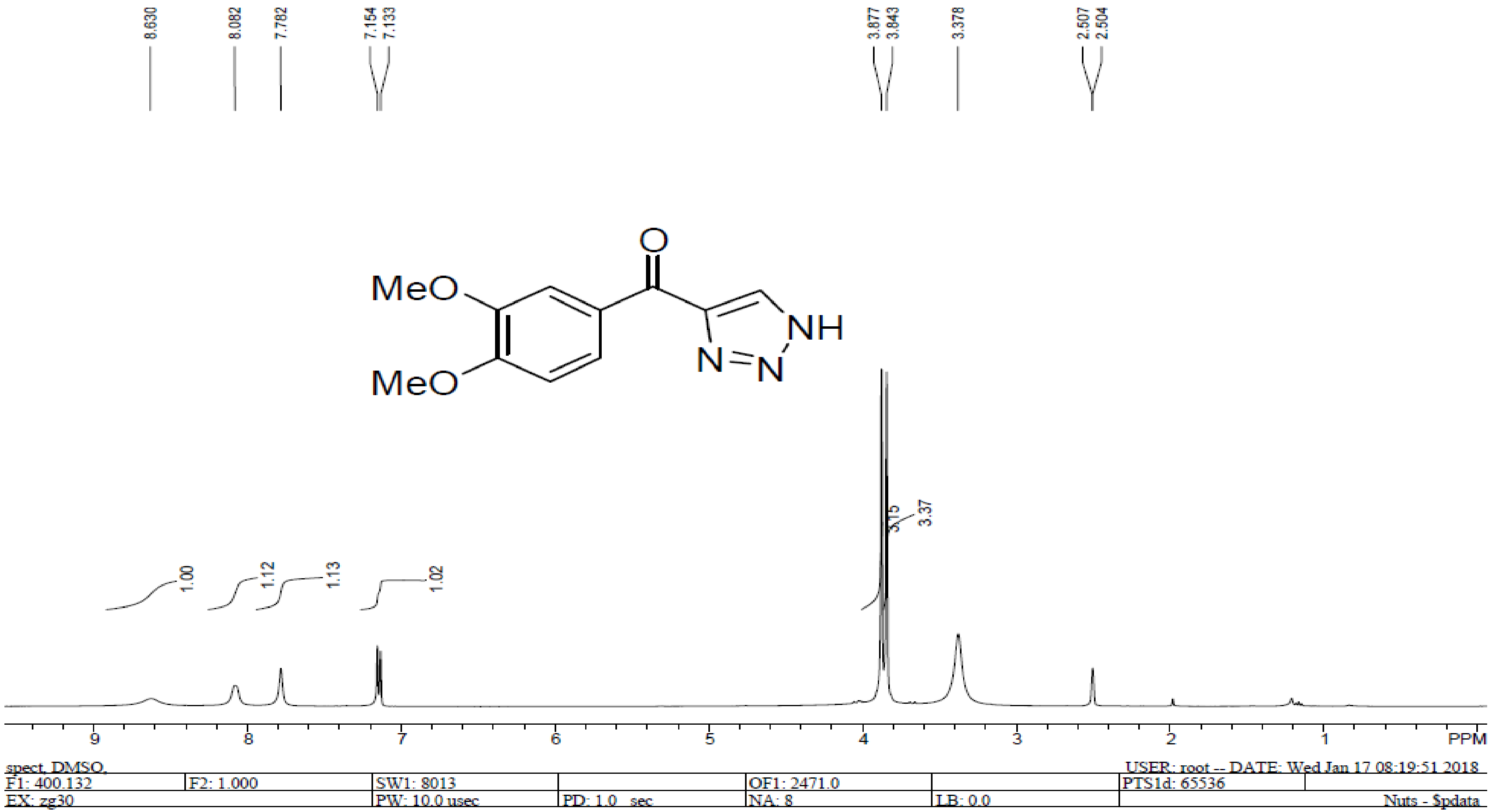

^{1}H NMR spectrum of **3p**

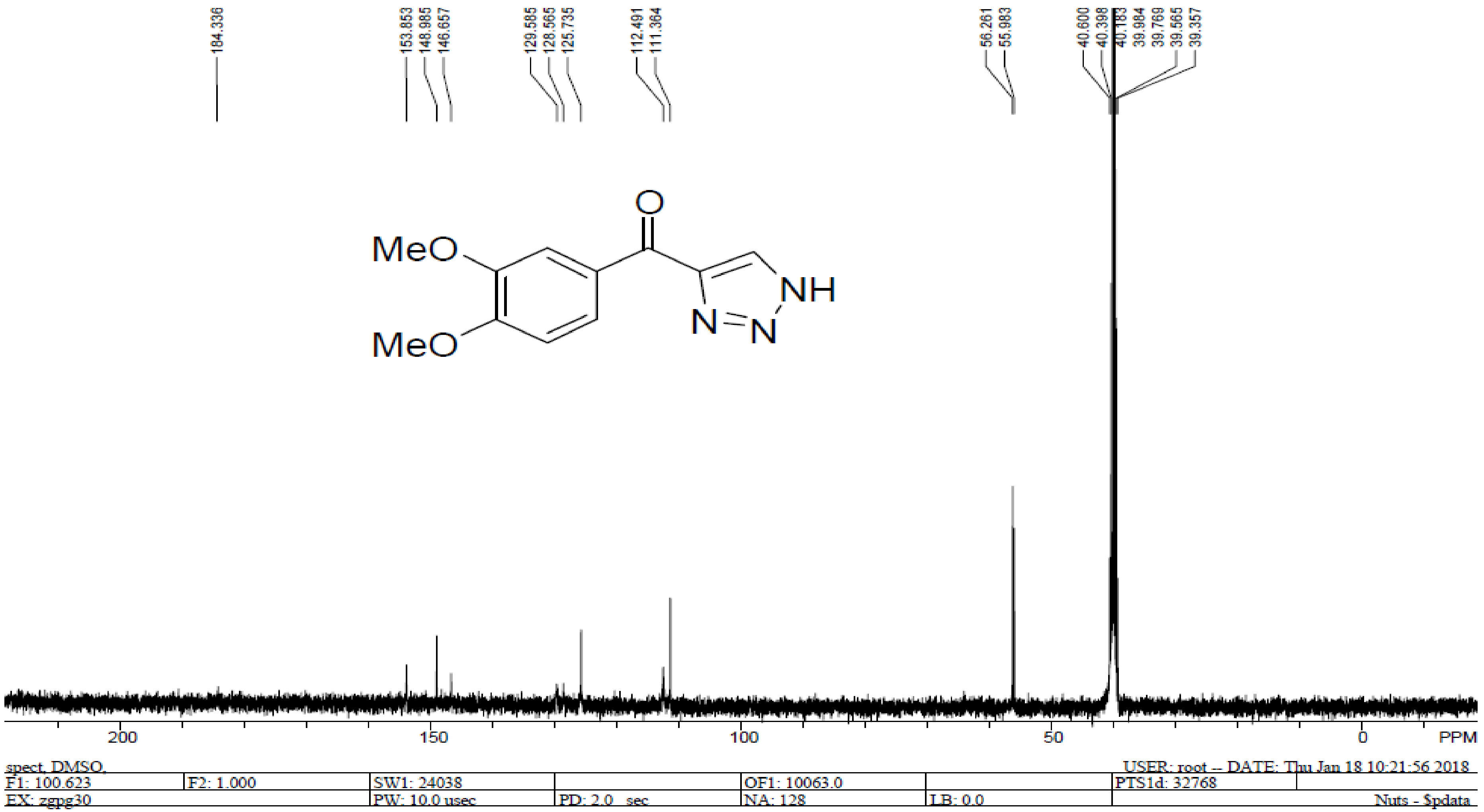

^{13}C NMR spectrum of **3p**

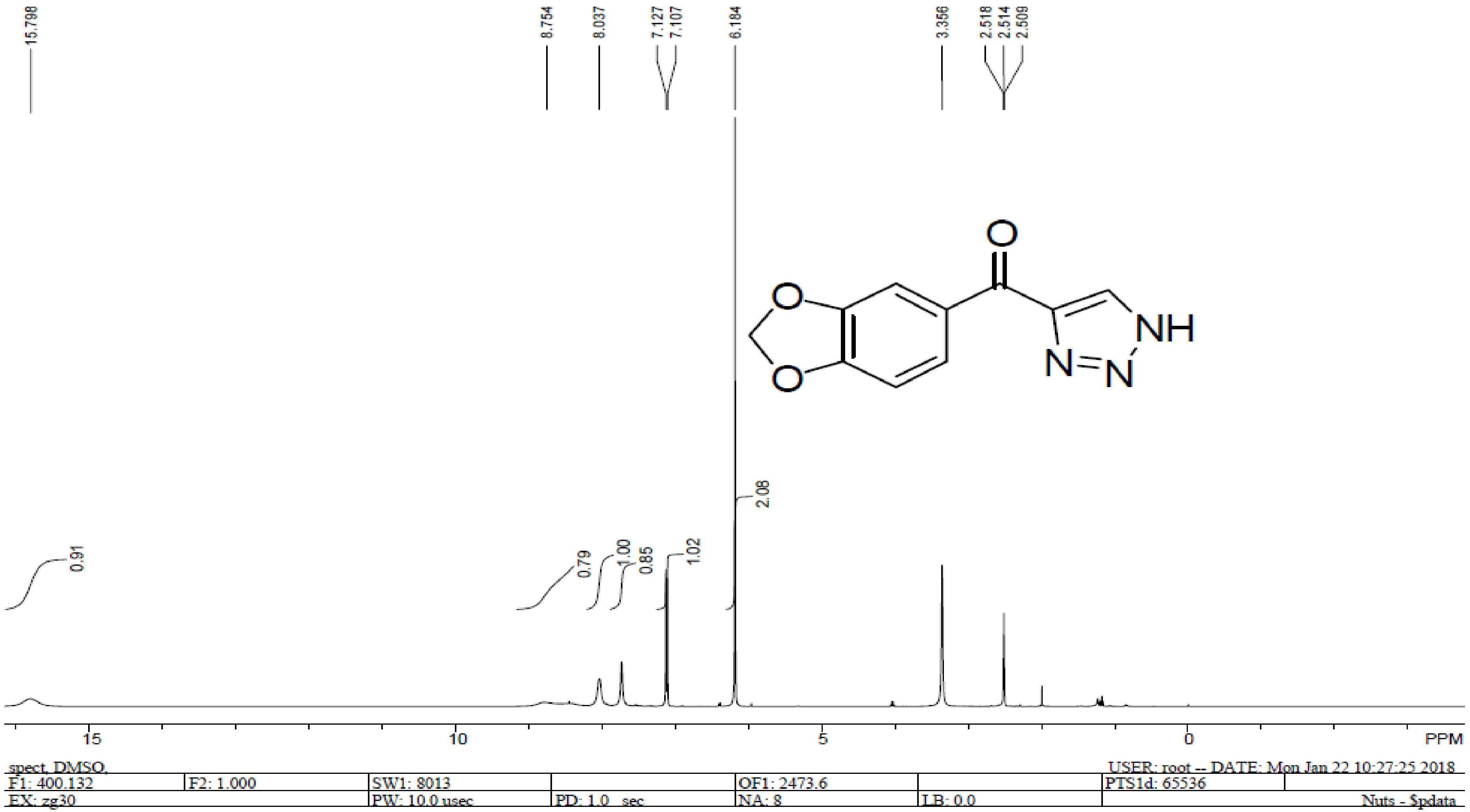

^{1}H NMR spectrum of **3q**

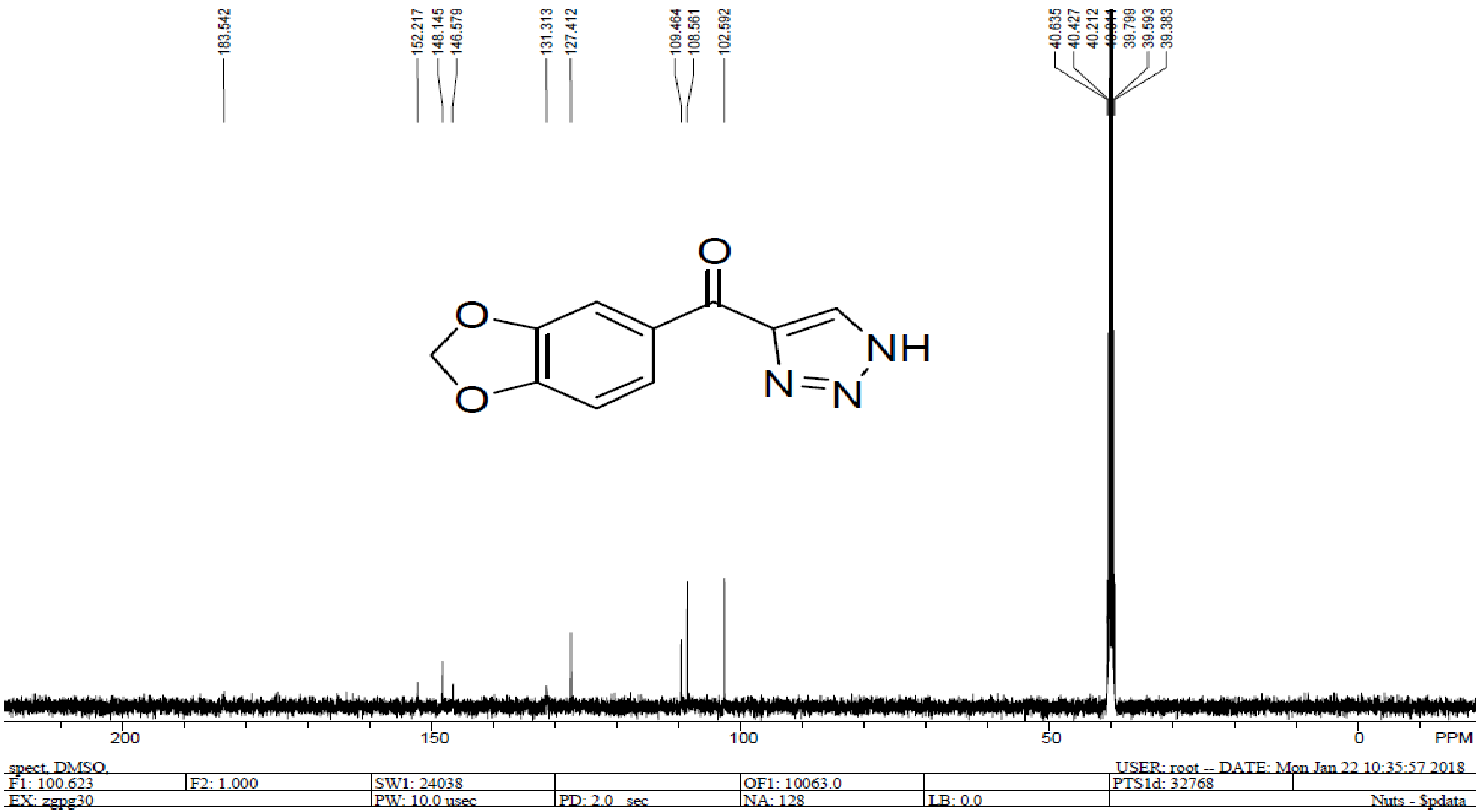

^{13}C NMR spectrum of **3q**

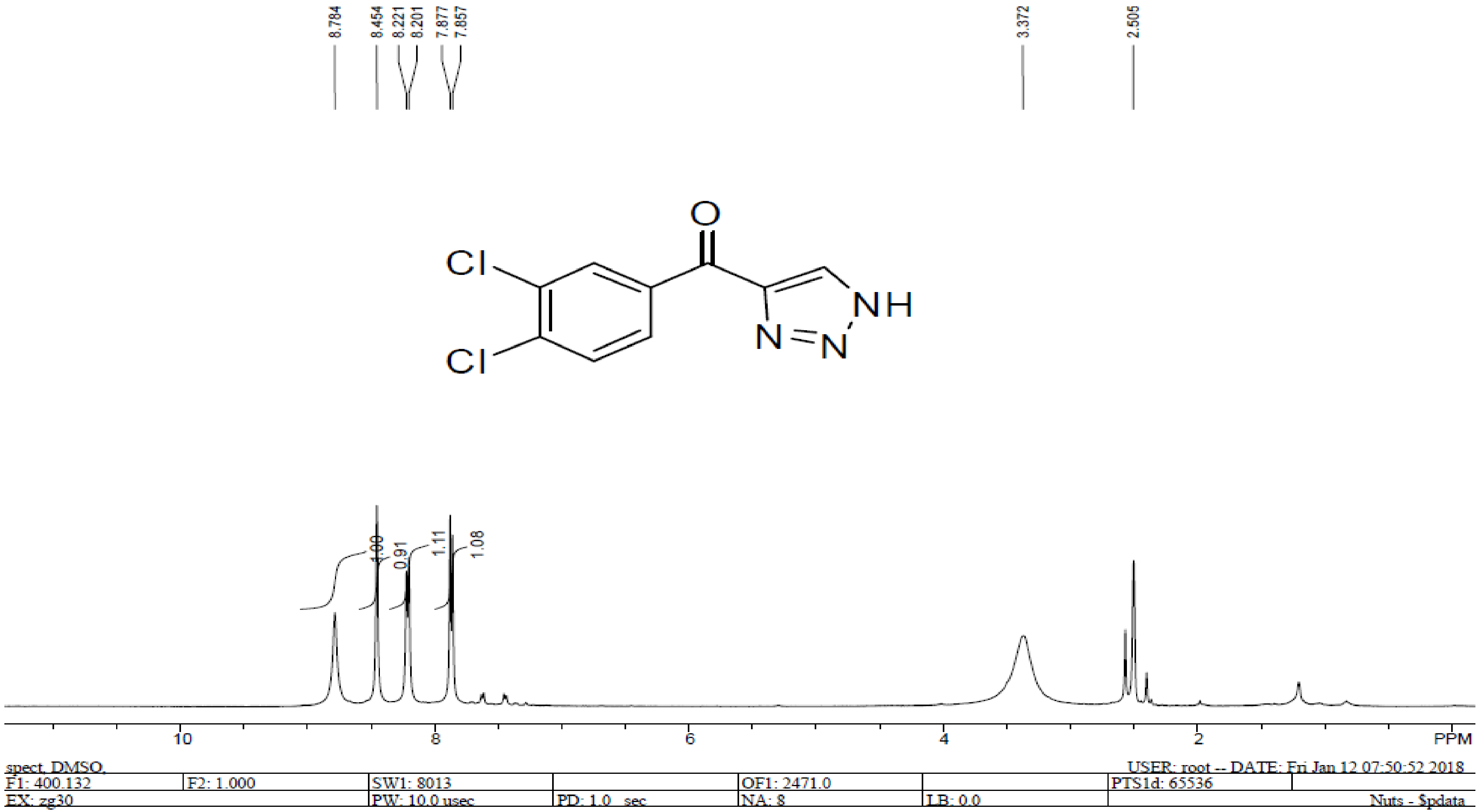

^{1}H NMR spectrum of **3r**

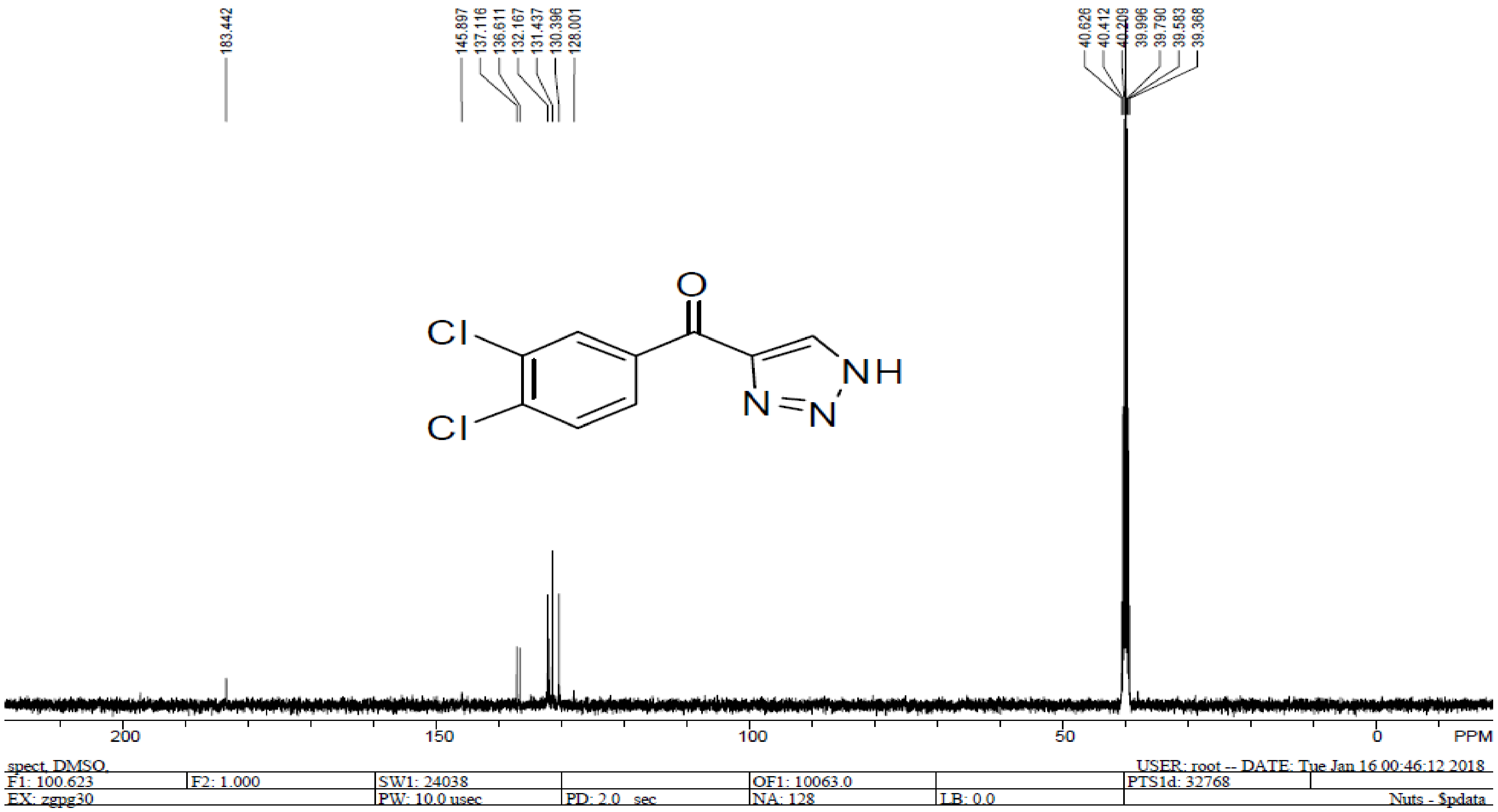

^{13}C NMR spectrum of **3r**

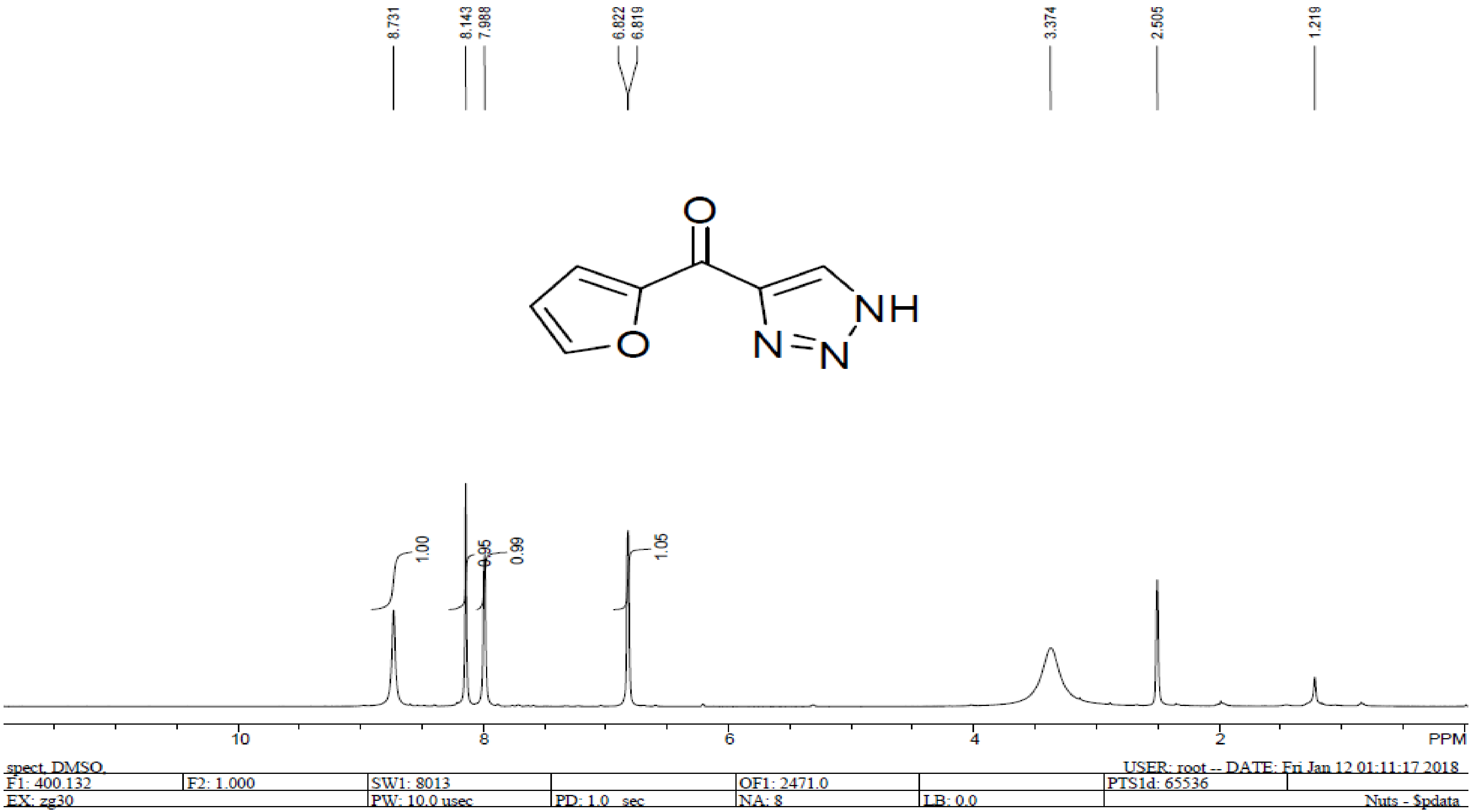

^{1}H NMR spectrum of **3s**

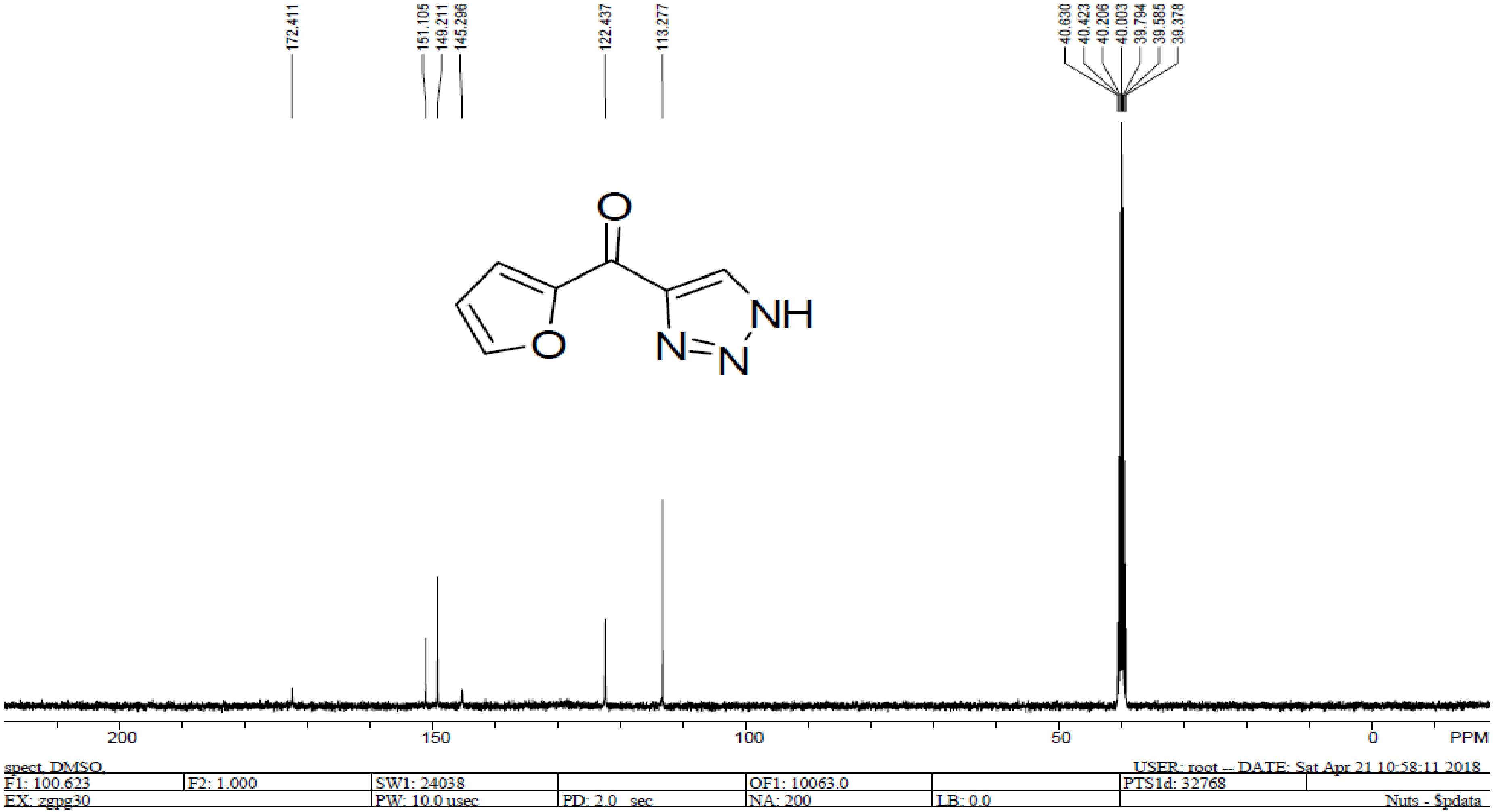

^{13}C NMR spectrum of **3s**

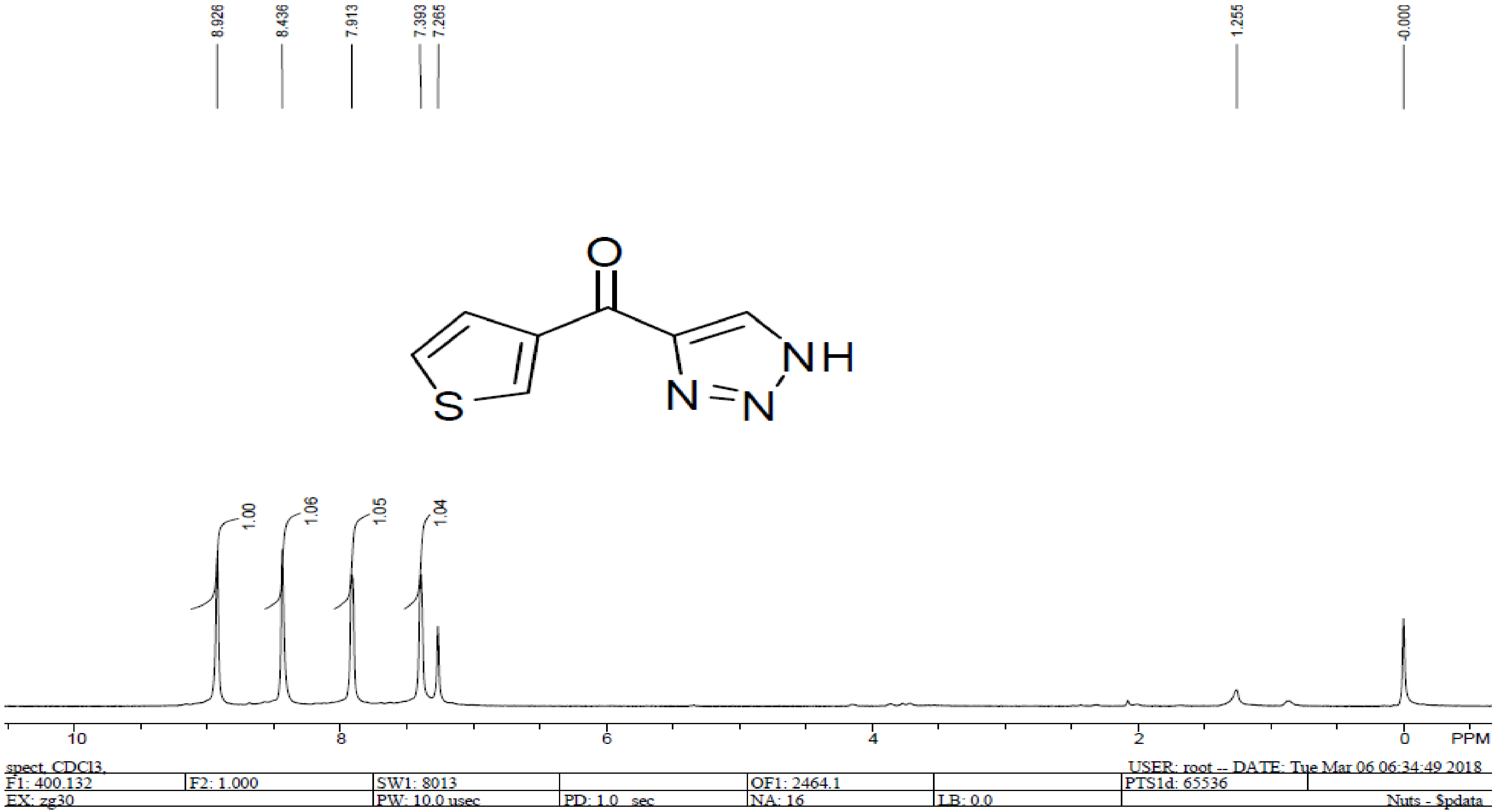

^{1}H NMR spectrum of **3t**

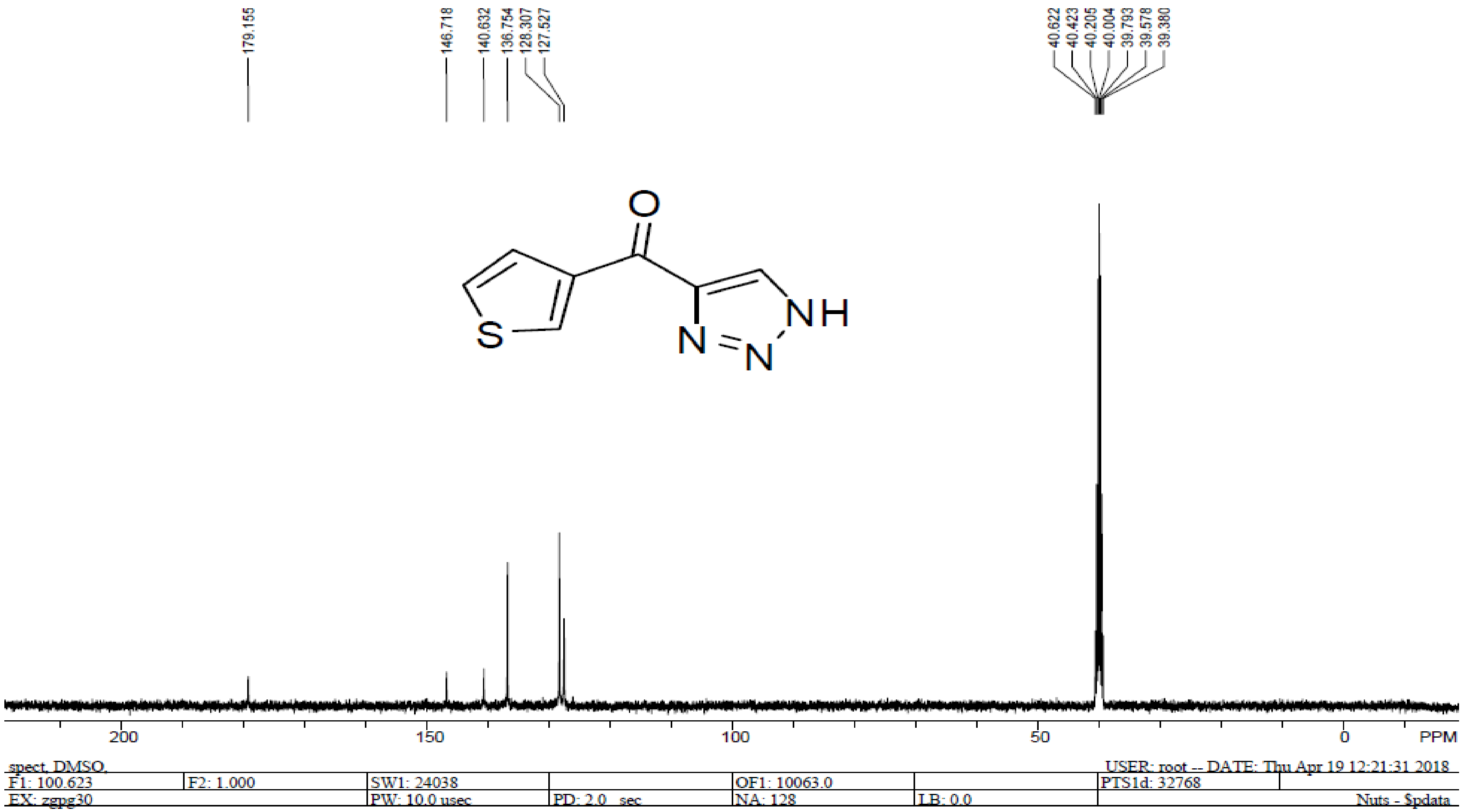

^{13}C NMR spectrum of **3t**
